# Current Landscape in Organic Nanosized Materials Advances for Improved Management of Colorectal Cancer Patients

**DOI:** 10.3390/ma14092440

**Published:** 2021-05-08

**Authors:** Octav Ginghină, Ariana Hudiță, Cătălin Zaharia, Aristidis Tsatsakis, Yaroslav Mezhuev, Marieta Costache, Bianca Gălățeanu

**Affiliations:** 1 Department of Surgery, “Sf. Ioan” Emergency Clinical Hospital, 13 Vitan Barzesti Street, 042122 Bucharest, Romania; octav.ginghina@umfcd.com; 2 Department II, Faculty of Dental Medicine, “Carol Davila” University of Medicine and Pharmacy Bucharest, 17-21 Calea Plevnei Street, 010232 Bucharest, Romania; 3 Department of Biochemistry and Molecular Biology, University of Bucharest, 91-95 Splaiul Independentei Street, 050095 Bucharest, Romania; marieta.costache@bio.unibuc.ro (M.C.); bianca.galateanu@bio.unibuc.ro (B.G.); 4 Advanced Polymer Materials Group, Department of Bioresources and Polymer Science, University Politehnica of Bucharest, 1-7 Gh. Polizu Street, 011061 Bucharest, Romania; zaharia.catalin@gmail.com; 5 Department of Toxicology and Forensic Sciences, Faculty of Medicine, University of Crete, 71003 Heraklion, Greece; tsatsaka@uoc.gr; 6 Center of Biomaterials, D Mendeleev University of Chemical Technology of Russia, Miusskaya Sq. 9, 125047 Moscow, Russia; valsorja@mail.ru

**Keywords:** drug-delivery systems, nanoparticles, liposomes, colorectal cancer, target therapy, nanomedicine

## Abstract

Globally, colorectal cancer (CRC) ranks as one of the most prevalent types of cancers at the moment, being the second cause of cancer-related deaths. The CRC chemotherapy backbone is represented by 5-fluorouracil, oxaliplatin, irinotecan, and their combinations, but their administration presents several serious disadvantages, such as poor bioavailability, lack of tumor specificity, and susceptibility to multidrug resistance. To address these limitations, nanomedicine has arisen as a powerful tool to improve current chemotherapy since nanosized carriers hold great promise in improving the stability and solubility of the drug payload and enhancing the active concentration of the drug that reaches the tumor tissue, increasing, therefore, the safety and efficacy of the treatment. In this context, the present review offers an overview of the most recent advances in the development of nanosized drug-delivery systems as smart therapeutic tools in CRC management and highlights the emerging need for improving the existing in vitro cancer models to reduce animal testing and increase the success of nanomedicine in clinical trials.

## 1. Introduction

Globally, colorectal cancer (CRC) ranks as one of the most prevalent types of cancers at the moment, being the second cause of cancer-related deaths [1,2]. According to World Health Organization (WHO), CRC is positioned worldwide as the third most common type of cancer as incidence, and the second as mortality, with 1.93 million new cases and 935,000 deaths reported in 2020 [2,3]. The CRC incidence is directly correlated with the economic status of the countries, the most affected countries being low- and middle-income, where lifestyle habits and dietary patterns sustain the development of this malignancy [4,5,6]. Despite the existence of screening programs that allow CRC diagnosis in early stages [7], CRC morbidity and mortality are steadily increasing even in developed countries. This trend is correlated with the disease stage at the time of diagnosis that holds a great impact on the 5-year relative survival rate of CRC patients. The 5-year relative survival rate decreases from ~90% for stage I CRC to ~10% for stage IV metastatic CRC (mCRC) [8], the disease stage being also a crucial factor in the establishment of a proper and effective therapeutic approach. Unfortunately, the onset of CRC is asymptomatic, a feature that is responsible for the large number of patients in advanced stages of the disease at initial diagnosis. While one-quarter of the CRC patients are diagnosed from the beginning with mCRC, more than half of the patients will develop metastasis along with CRC progression [1,9]. More, the initiation of the screening programs is typically recommended for adults with ages above 50 years, but the recent rising incidence among young adults could rewrite the current CRC screening guidelines [10]. For example, the American Cancer Society already lowered the age threshold to 45 years for individuals with risk for CRC development [11], but further adjustments need to be done at a global level to lower the CRC burden and ensure the detection of CRC in early stages. 

At the moment, several therapeutic approaches are implemented in clinics for CRC management such as surgery [12,13,14], radiotherapy [15,16], chemotherapy [17,18,19], and targeted therapy if applicable [20]. The choice of the approach is dependent on the stage of CRC at the initial diagnosis. While CRC patients diagnosed in the early stages of the disease by colonoscopy or sigmoidoscopy, which present a clean tumor with well-delimited margins, qualify for surgical resection of the tumor, the major challenge is represented by patients in stage III/IV, where the disease is spread to the lymph nodes and distant organs. For these patients, adjuvant chemotherapy is mandatory to control CRC and metastatic invasion, followed by tumor and metastases surgical resection if allowed [21]. 

Despite the recent advances in cytotoxic chemotherapy and targeted therapy, the prognosis of CRC remains unsatisfactory, especially for mCRC patients [22]. The severe side effects associated with chemotherapy and the development of multidrug resistance are critical issues that hinder the proper care of CRC patients by limiting treatment efficacy and leading to chemotherapy failure. In this view, nanomedicine has emerged as a powerful tool to improve the existing drug-based strategies for CRC treatment. Nanomedicines are nanosized carrier biomaterials that are used as shuttles to deliver drug cargos to the tumor tissue. These nanosized drug-delivery systems possess the potential to improve the stability and solubility of the drug payload and to enhance the active concentration of the drug that reaches the tumor tissue, increasing the safety and efficacy of the treatment [23,24]. In this context, the present review offers an overview of the most recent advances in the development of organic nanosized drug-delivery systems as smart therapeutic tools in CRC management. 

## 2. Current Pharmacotherapy Available for CRC

**Figure 1:**
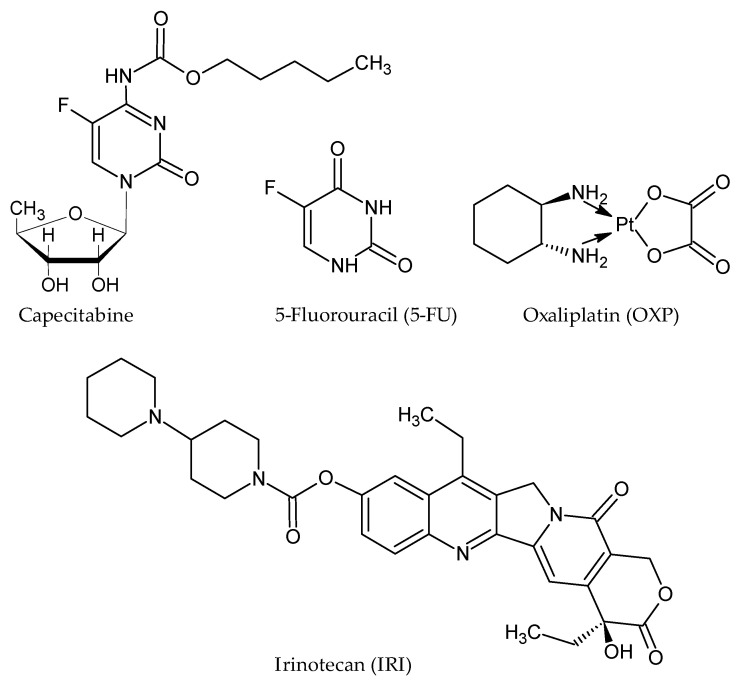
Structure of conventional chemotherapeutic agents administrated in CRC.

The existing pharmacotherapy available for CRC treatment relies on the administration of cytotoxic agents, targeted therapies, and their combinations. Among cytotoxic agents, CRC chemotherapeutic regiments are based on the administration of capecitabine or 5-fluorouracil (5-FU), oxaliplatin (OXP), and irinotecan (IRI) (Figure 1), anticancer agents that are usually combinatorically administrated to amplify their antineoplastic potential: 5-FU/LV/OXP (FOLFOX), 5-FU/LV/IRI (FOLFIRI), and 5-FU/LV/IRI/OXA (FOLFIRINOX) [25,26]. These anticancer agents act on both DNA or RNA synthesis to exert their cytotoxic effects [27,28,29], and although they still represent the backbone of CRC chemotherapy, their administration presents several serious disadvantages, such as poor bioavailability [27,30,31], lack of tumor specificity, and susceptibility to multidrug resistance [28,32,33]. 

One such example is 5-FU, a fluoropyrimidine analogue and a first-line drug in colorectal cancer chemotherapeutic regimens. The conversion of 5-FU into its inactive metabolite, 5,6-dihydro-5-fluorouracil (DHFU), is directly dependent on the dihydropyrimidine dehydrogenase (DPYD) enzyme, which is encoded by the DPYD gene [34]. Several genotypic variants of DPYD were identified in the population that can be translated into individual variations in the drug’s clearance [35], leading to 5-FU systemic toxicity and drug resistance [36]. Consequently, 5-FU administration is associated with side effects that can vary from mild to severe, depending on the patient’s tolerance [37,38], severely limiting the dose of the drug and leading to poor antineoplastic results.

For a more specific approach to CRC pathology, the strategy relies on targeting molecules that play a central role in tumor development and progression, generally by using monoclonal antibodies [39]. In this respect, anti-angiogenic drugs targeting the vascular endothelial growth factor (VEGF) pathway hold a key role in treating patients with metastatic CRC. Bevacizumab [40,41,42], ramucirumab [43], and aflibercept [44,45] are used for targeting angiogenesis, a culprit in tumor development and progression [46,47]. For CRC patients that do not harbor KRAS or NRAS mutations, cetuximab and panitumumab can be administrated as anti-EGFR antibodies [48,49,50]. Epidermal growth factor receptor (EGFR) modulates CRC initiation and progression due to its key role in activating downstream signaling pathways that control tumor cell growth, differentiation, and proliferation [51,52,53,54]. More recently, immune checkpoint blockade agents such as pembrolizumab, nivolumab, and ipilimumab have been exploited as targeted therapy in CRC [20]. Promising clinical studies raise the hope of survival improvement in CRC patients when administering bevacizumab and standard chemotherapy combined approach [55,56].

## 3. Targeting Strategies for Drug-Delivery Systems in CRC

An ideal drug-delivery system for anticancer agents should be designed and developed to significantly improve the efficacy of the drug-free traditional treatment by functioning as a protective shuttle for avoiding drug degradation, thus ensuring an increased drug concentration that reaches the tumor. More, in the preparation phase, the nanoparticles should be tailored to enhance their tumor selectivity and ensure accumulation to the tumor site, reducing therefore the cytotoxic effects on normal healthy tissues. 

The physicochemical proprieties of the nanocarriers impact their effectiveness as drug-delivery systems in CRC management. The size and shape of the nanoparticles, as well as the charge of their surface, have a non-specific effect, which is determined by the interaction with the cell membrane [57]. Since the surface of the cell membrane is negatively charged, positively charged particles have the highest penetration rate, while the lowest penetration rate is observed at nanoparticles with a negative surface charge [58]. It has been shown that a high positive charge on the surface of particles leads to enhanced cellular uptake, while a negative charge contributes to reduced absorption of nanoparticles [59]. It is noteworthy that the immobilization of some proteins on the surface of charged nanoparticles has a leveling effect on the charge factor, contributing to a similar rate of absorption of positively and negatively charged particles [60].

The size of nanoparticles has a significant impact on the rate and mechanism of their absorption. The rate of nanoparticle absorption by cells increases with particle size [61]. For particles with a diameter of more than 200 nm, absorption generally occurs through clathrin-dependent endocytosis. The mechanism of smaller particle absorption remains a subject of discussion, being probably associated with passive transport through the pores of the cell membrane or directly through membrane fusion [62]. Lastly, the nanoparticles smaller than 100 nm may be absorbed through the nuclear membrane, enabling the transport of drugs into the cell nucleus. Thus, the size of the carrier particles determines the rate and mechanism of its absorption by the cell, as well as the character of drug distribution between the cell organelles [63]. 

The penetration rate of nanoparticles into the cell also depends on their geometric shape. It has been shown that spherical particles are absorbed by cells at the highest rate [64]. The interpretation of data on the effect of the size and shape of particles on the nature of their absorption by the cell should be carried out with caution since nanoparticles are simultaneously involved in at least three processes: diffusion, sedimentation, and agglomeration [61]. 

**Figure 2:**
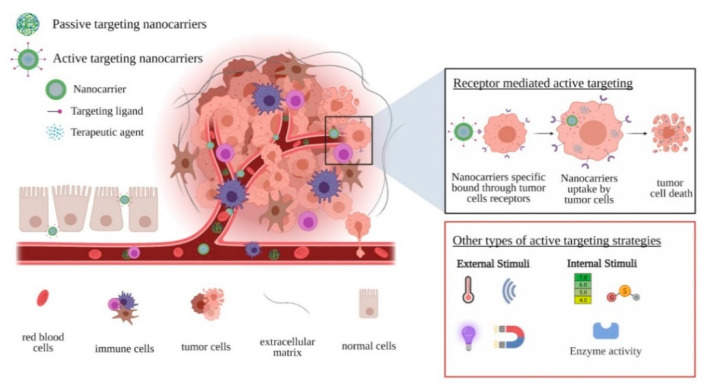
Schematic representation of drug-delivery systems targeting strategies.

Although these factors are common for the therapy of all types of cancers, CRC of various etiology [65,66] has special requirements for ensuring the bioavailability of pharmacologically active substances delivered through nanoparticles [67]. The problem of increasing the bioavailability of drugs can be solved, at least partially, by introducing CRC-specific ligands on nanoparticle surfaces [68,69]. Based on these observations, there are two strategies by which nanosized drug-delivery systems reach tumor cells (Figure 2): (i) passive targeting, where nanoparticles take advantage of the abnormalities of tumor vasculature to accumulate at the tumor site, and (ii) active targeting, where nanoparticles are functionalized with moieties that directly target the tumor cells.

### 3.1. Passive Targeting

The passive targeting strategy is based on the capacity of the nanosized drug-delivery systems to passively accumulate in tumor cells, due to the enhanced permeability and retention (EPR) effect. The development of tumors relies on angiogenesis as a requisite for tumor growth and metastasis, as the tumors fail to develop over a few mm in the absence of a supportive vasculature network that supplies oxygen and nutrients [70,71,72]. This newly formed tumor vascular network is characterized by a disorganized architecture, hyperpermeability, impaired blood flow, and loose interconnections between endothelial cells [73,74,75,76]. Due to the high permeability of tumor blood vessels and their impaired lymphatic drainage system, nanocarriers reach the tumor through preexisting vascular endothelial gaps and accumulate in the tumor tissue, where are retained for prolonged times in the absence of a functional lymphatic system [77], a phenomenon called the EPR effect. As the drug-delivery systems take favor of the leaky tumor blood vessels to reach tumors, the nanocarrier size is an important characteristic in passive targeting. The tumor vascular pore diameter measures between 100 nm to 2 μm depending on the cancer type and stage, but in CRC, sizes usually range between 400 and 600 nm [78,79]. This aspect should be taken into consideration in drug-delivery system development, since the size mediates the ease by which the tumor tissue captures the nanocarriers from circulation, as the nanoparticles need to fit the existing endothelial fenestrations to reach tumors. It is considered that the ideal particle size developed should be in the range of 10–200 nm, as it is considered that in this range the particles lack the ability to extravasate normal tissues and avoid clearance mediated by renal excretion and phagocytosis [80,81]. 

On the other hand, the fate of the carriers upon administration needs to be taken into consideration in the nanosized drug-delivery system design. Once in circulation, nanoparticles interact with the host immune system that recognizes them as foreign objects and activates defense mechanisms. As a result, nanocarriers are engulfed by cells of the mononuclear phagocyte system (MPS) and are rapidly cleared from the systemic circulation before reaching the tumor tissue [82,83]. Therefore, conventional nanosystems need to be tuned to improve their in vivo stability and to avoid their rapid systemic clearance by the MPS [84]. In this view, the most popular choice for shielding nanocarriers is PEGylation, but other strategies can be also employed to achieve long-circulating drug-delivery systems [85]. Polyethylene glycol (PEG) is a versatile polyether diol that is attractive for biomedical applications due to its excellent biocompatibility and solubility and low immunogenicity [86,87]. PEG acts as a protective hydrophilic film that helps nanoparticles to escape the MPS cells and to avoid aggregation [88,89]. Even if PEGylation is intensively used for nanosized systems camouflage and has proven to be a functional strategy for improving circulation stability and nanoparticle accumulation in tumors, the use of this approach remains controversial for gene and nucleic acid delivery. The presence of the PEG aqueous coating on the liposome surface inhibits the interaction of the nanocarriers with the cancer cell surfaces and receptor-mediated endocytosis, leading therefore to a severely reduced cellular uptake [90,91]. In this context, strategies for overcoming this so-called “PEG dilemma” should be employed for the prospective use of nanomaterials for drug-delivery of low-dosage therapeutic cargos such as pDNA, siRNA, or miRNA.

In conclusion, the ideal passive targeting nanoshuttle should meet the following two properties: (i) appropriate particle size for efficient pass-through the tumor neovasculature, and (ii) “invisibility” against the host immune system.

### 3.2. Active Targeting

The discovery of specific molecular aberrations on colorectal tumor cells surface has opened the opportunity for the development of superior nanomedicines by enriching the nanosized drug-delivery system surfaces with different moieties that specifically bind to receptors that are overexpressed in colorectal cancer. This strategy helps the nanoparticles to discriminate between healthy and tumor cells, thus improving tumor specificity and ensuring a superior cellular uptake of nanoparticles into tumor cells. Different moieties such as monoclonal antibodies, aptamers, nucleic acids, small molecules, and peptides are widely used for nanoparticle surface functionalization and to further assist tumor cell localization and uptake. 

Transferrin receptors (TFRs) are membrane glycoproteins involved in iron transport through transferrin binding and receptor-mediated endocytosis, which are expressed at low levels in the majority of normal tissues [92,93]. Iron is vital for cell proliferation, and due to the increased need of tumors for nutrients, including iron, the overexpression of TFR1 is identified in cancer patients including CRC [94,95]. While the abundance of TFR1 impacts colorectal cancer cell proliferation rates [96], this is particularity appealing for engineering nanoparticles in CRC-targeted therapy by surface functionalization with TFR’s natural ligand transferrin [97,98,99,100]. 

Folate receptors (FRs) are membrane-bound glycoproteins that show a high affinity for folates and folate conjugates [101] and are overexpressed in epithelial cancers [102]. FRs sustain tumor cell proliferation, migration, and invasion, and their levels impact the CRC patient’s average life expectancy [103,104]. FRs bind folate, a water-soluble vitamin involved in amino acid metabolism and DNA and RNA synthesis [105], with a vital role in cell survival and proper development. In cancers, the overexpression of FRs leads to an increased intracellular concentration of folate, a nutrient that sustains the rapid and uncontrolled tumor cell proliferation [106,107]. Folate enrichment of nanoparticles can increase the selectivity of the drug-delivery systems for colorectal tumor cells, improving, therefore, therapy efficacy [108,109,110,111,112,113,114]. 

Hyaluronic acid (HA) is a popular molecule for tailoring nanocarriers to target CD44, a transmembrane glycoprotein overexpressed in cancers, with implications in tumor cell proliferation, differentiation, motility, and chemoresistance [75,115]. More, CD44 is part of the molecular signature of cancer stem cells, a subpopulation of cells involved in tumor initiation, growth, and metastasis [116,117]. Therefore, HA-decorated nanoparticles could target both tumor cells overexpressing CD44 as well as cancer stem cells [118,119,120,121,122]. 

Antibodies are also widely used for nanocarrier functionalization based on their inherent capability to specifically recognize their targets, and the promising results of cetuximab and panitumumab administration in improving clinical outcomes for mCRC patients lead to the use of these molecules as functionalization agents to direct nanoparticles towards tumor cells that overexpress EGFR. Several anti-EGFR-coated nanoparticles loaded with 5-FU were synthesized for CRC applications and showed superior colorectal cancer cell specificity and cytotoxicity of the novel drug-delivery systems [123,124,125]. An alternative for antibody use are aptamers, small single-stranded DNA or RNA molecules that present superior binding specificity and affinity for targeted molecules and low immunogenicity [126]. Yao et al. used a self-assembled DNA-nanocross functionalized by four AS1411 aptamers for doxorubicin delivery to target the overexpressed nucleolin localized on the colorectal tumor cell surface and found that the drug-delivery system developed was selectivity delivered to colorectal carcinoma cells and presented an enhanced antitumor efficacy compared to free doxorubicin [127]. 

Another approach explored for active targeting purposes is the use of stimuli-responsive nanocarriers that release their drug payload as a response either to intrinsic stimuli provided by the pathological microenvironment or to extrinsic stimuli delivered artificially. In the case of endogenous stimuli-responsive nanosystems, the release of the encapsulated drug cargo from nanoparticles is triggered by tumor-specific environmental clues (pH, hypoxia, redox potential, enzymes) [128]. A common choice is represented by pH-responsive nanocarriers developed based on the clear differences in pH observed between tumor (pH < 6.5) and normal tissues (pH 7.4), which are generated by marked acidification at the tumor site as a result of excessive glycolysis and impaired waste removal mechanisms [129,130]. For exogenous stimuli-responsive nanosystems, various external stimuli (light, thermal, magnetic, ultrasound) can be provided to achieve the nanoparticles’ therapeutic cargo-specific discharge at the tumor site [128]. Examples of such nanosized particles engineered as stimuli-responsive drug-delivery systems in CRC are summarized in Table 1. 

## 4. Organic Nanosized Drug-Delivery Systems for CRC Therapy

**Figure 3:**
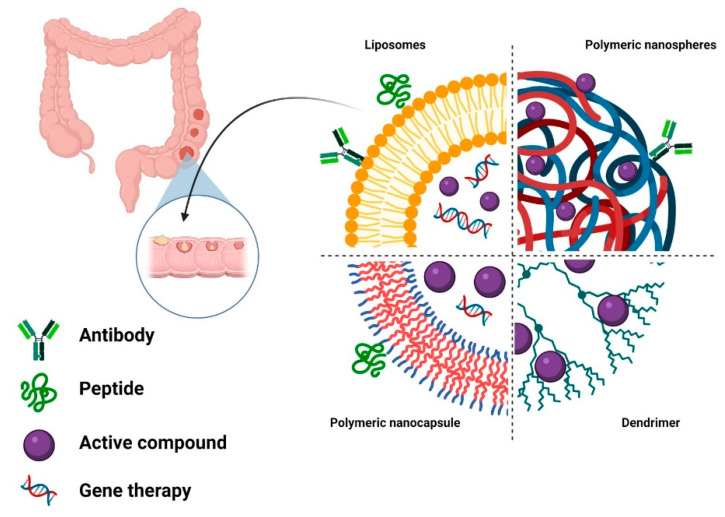
Main types of organic nanosized drug-delivery systems.

As previously discussed, both conventional and targeted chemotherapies display toxic side effects related to the unspecific mechanism of action, elevated blood levels, and high toxicity. Targeting specific tumor cells without exposing the normal cells to the drugs might be a promising strategy to scale down the administration dose. One of the landmarks in this respect is the development of nanosized drug-delivery systems (Figure 3), which hold great promise to enhance the therapeutic efficacy and safety profile of the conventional agents. These nanoshuttles are designed to deliver antitumor agents at the tumor site either by taking benefit from the tumor pathophysiology or by actively targeting the tumor cells.

The development of the nanosized drug-delivery systems holds great promise not only for the decrease of conventional chemotherapeutics’ systemic toxicity and for enhancement of their bioavailability, but also for transporting novel therapeutics such as genes or proteins. For a more modern approach, nanosized drug-delivery systems are also used for the delivery of novel therapeutics, such as siRNA and peptides/proteins. siRNAs and miRNAs, considered next-generation cell-pathway inhibitors, are novel approaches in CRC management, targeting the aberrant alteration of certain specific gene expressions. Currently, it is widely accepted that the accumulation of mutations may induce the overexpression of oncogenes with subsequent inactivation of tumor suppressor genes that trigger functional alterations in proteins controlling cell division, apoptosis, the cell cycle, etc. [137]. Due to their chemical nature, siRNAs and microRNAs are susceptible to quick degradation and poor intracellular uptake, and therefore can be encapsulated in nanoformulations such as polymers, micelles, nanovectors, liposomes, etc. For example, the encapsulation of CXCR4-siRNA in dextran–spermine polymer micelles can reduce the expression of CXCR4 protein, decreasing the rate of liver metastasis in CRC [138], while the delivery of miR-20a through chondroitin sulfate–sorbitan ester nanoparticles mediated restoration of normal mir-20a levels with the impact of the expression of its protein targets [139].

### 4.1. Lipid-Based Drug-Delivery Systems 

Among lipid-based drug-delivery systems, liposomes are the most extensively used and well-characterized drug carriers. First described in the mid-1960s [140], liposomes are currently a promising tool for cancer nanomedicine, being the first nanosized drug-delivery systems that have been successfully implemented for clinical use in breast, ovarian, or pancreatic cancer therapy [141,142]. With a structure that mimics the native cell membrane, liposomes are small spherical vesicles consisting of one or more concentric lipid bilayers bounding an aqueous compartment. This unique structure sustains the development of liposomal formulations as carriers for both hydrophilic and hydrophobic drugs [143] and also enhances the cellular uptake process [144], liposomes being easily incorporated into cells by absorption, phagocytosis, or fusion [145]. Moreover, liposomes show excellent biocompatibility and biodegradability, and low immunogenicity [146]. These lipid-based drug vehicles can be simply obtained based on cholesterol and natural phospholipids and easily tailored in terms of size, composition, charge, and lamellarity. Different compositions employed for liposome synthesis are presented in Table 2.

Several liposomal formulations of CRC traditional chemotherapeutic agents were developed to improve the current treatment efficacy and reduce the severe side effects associated with the administration of the free drugs. The majority of these liposomal formulations are obtained by the thin layer film hydration method, which is the most commonly used protocol for liposome generation [151,152]. Additionally, different nanosizing techniques are employed to modulate the liposome size, which further impacts liposome clearance and accumulation in the tumor tissue: extrusion, ultrasonication, freeze–thaw sonication, and homogenization [153]. 

A 5-FU liposomal formulation was prepared by the classic thin layer film hydration method [140] to minimize the free-drug toxicity and increase its anticancer activity. Mansoori et al. [118] prepared 5-FU loaded liposomes functionalized with HA to selectively target CD44 overexpressing tumor cells and showed the superior potential of high CD44 HT-29 adenocarcinoma cells to internalize 5-FU HA liposomes as compared with low CD44 HepG2 cells. Overall, the 5-FU HA liposomes presented good cytotoxicity against colorectal cancer cells, triggered cell-cycle arrest in G0/G1 phases and tumor cell apoptosis, and suppressed HT-29 colony-forming capacity, showing a superior in vitro antitumoral potential as compared with 5-FU liposomes with non-functionalized surfaces. The same colorectal cancer in vitro model was employed to reveal the cytotoxic and proapoptotic effects of 5-FU loaded transferrin liposomes [100]. The 5-FU transferrin liposomes displayed low cytotoxic effects on fibroblasts cells and an enhanced potential in activating the mitochondrial apoptosis signaling as compared with free 5-FU. A similar mechanism of action was identified for folate-liposomal 5-FU in HeLa cells, while in HT-29 cell cultures this liposomal formulation triggered cell necrosis and ROS overproduction [147]. The cytotoxic potential of the folate-liposomal 5-FU was superior for all screened tumor cell lines in comparison with free 5-FU and 5-FU non-functionalized liposomes and was minimal on normal cells, while in vivo experiments revealed an augmented decrease of the tumor volume after exposure to 5-FU folate liposomes vs. free 5-FU. 

Chen and colleagues [148] designed another strategy for improving 5-FU treatment efficacy in CRC by developing, through the ethanol injection method, rapamycin loaded liposomes that can be administrated either alone due to the anticancer activity [154,155] of rapamycin or assist the free 5-FU treatment to potentate its cytotoxic activity. The obtained results showed that the cellular uptake, cytotoxic, and apoptotic potential were enhanced following liposomal encapsulation of free rapamycin. Combining the liposomal rapamycin with free 5-FU triggered a synergistic antitumor effect via the Akt/mTOR and P53 pathways.

For IRI delivery, several liposomal formulations have been developed and exhibited superior anticancer potential when compared with the free drug, and many of these formulations encapsulate directly IRI’s active metabolite SN-38 [132,149,156,157,158,159]. For example, Xing et al. [149] developed, by the ethanol injection method, stable liposomal carriers of moeixitecan, a lipophilic SN38 prodrug. Their outcomes depicted a superior cytotoxic activity and a significantly increased proapoptotic potential than free IRI and moeixitecan in HT-29 tumor cell cultures. In vivo results sustained the antitumoral potential of the liposomal moeixitecan that severely reduced tumor size in treated HT-29 colorectal xenograft models, while showing low toxicity on healthy tissues or blood components when compared with free IRI or moeixitecan administration. For OXP delivery, simple and PEGylated biomimetic magnetoliposomes were prepared by entrapping OXP biomimetic into liposomes. These formulations were hemocompatible and exhibited greater toxicity than OXP, with superior biocompatibility and cellular uptake exhibited by PEGylated nanosystems [150]. 

The observation that liposomes are unstable and exhibit leakage of encapsulated drugs led scientists to focus on another class of liposome-based drug-delivery systems, namely solid lipid nanoparticles, which are colloidal lipid particles with a solid lipid core at physiological temperature, with superior properties for stable drug entrapment and controlled release due to the solid-state of the lipid capsule. These nanoparticles show better physical stability than liposomes and no biotoxicity and can be employed for hydrophobic or poor-water soluble drug delivery [160]. These lipoformulations are fabricated by dispersing, in an aqueous solution containing surfactants, lipids that are solid at physiological temperature. Solid lipid nanoparticles show good biocompatibility, low toxicity, and ease of scale-up, but present a limited drug-loading capacity modulated by the solubility of the loaded drug in the lipid melt [161]. Therefore, a modified version of these nanosystems was generated by accommodating both solid and liquid lipids in the synthesis process, generating nanostructured lipid carriers that show an increased capacity for loading active pharmaceuticals than do classic simple lipid solid nanoparticles [162]. Smith et al. developed different 5-FU PEGylated solid lipid nanoparticles formulations that varied in lipid and surfactants composition and selected the most suitable formulation, based on HCT-116 tumor cell cytotoxic activity and efficiency of drug entrapment. The selected 5-FU PEGylated solid lipid nanoparticles show promising in vitro effects on HCT-116 cells and prolong plasma circulation of 5-FU. This 5-FU nanosystem triggered tumor growth suppression in tumor-bearing mice, with superior efficiency, compared to free 5-FU, by modulation of HER-2 expression, and further protected liver and kidney from 5-FU damage [163].

### 4.2. Polymer-Based Drug-Delivery Systems 

Based on the high diversity of polymers available for drug-delivery system development, numerous polymeric nanoparticles have been developed for cancer applications. The most common strategies for polymeric nanoparticle preparation are monomer polymerization and dispersion of preformed polymers [164], and a vast variety of natural and synthetic polymers or blends are available for nanoparticle synthesis. The polymer choice can impact the final proprieties of the developed nanocarriers and their biological performance. More, based on the materials employed in the synthesis protocol, polymeric nanocapsules, or nanospheres can be assembled, particles that vary in morphology, architecture, and drug loading strategies [165,166]. Nanospheres are constituted exclusively from polymers that form a solid spherical mass. In contrast, nanocapsules present a core–shell structure that implies the existence of a liquid/solid inner core surrounded by a polymeric membrane. In these polymeric nanoparticles, drugs can be entrapped, dispersed, dissolved within, or surface absorbed [167], but based on a superior capacity drug loading and enhanced protection of the drug payload, nanocapsules are preferred for drug-delivery applications rather than nanospheres. For nanocapsules, the core nature impacts the drug that can be entrapped and can be adjusted to fit the drug solubility. Therefore, nanocapsules can present an oily core or aqueous core to accommodate lipophilic or hydrophilic drugs, respectively [168,169]. The existence of a core within the nanocapsules is not mandatory, and hollow nanocapsules are being obtained using a solid spheric cast that can be removed by mild conditions after the polymeric shell is formed [170]. 

Regarding the polymer choice, polymeric nanoparticles can be obtained based on unique natural or synthetic polymers or from combinations. Due to their natural nature, polymers such as chitosan, collagen, gelatin, and silk fibroin present inherited appealing proprieties for nanoparticle synthesis, such as biodegradability, excellent biocompatibility, and ease of chemical modifications, and represent a more inexpensive choice for nanoparticle development [171]. Synthetic polymers such as poly(ethylene glycol) (PEG), poly(ε-caprolactone) (PCL), poly(glycolic acid) (PGA), poly(lactic acid) (PLA), poly(lactic-co-glycolic acid) (PLGA), or poly(3-hydroxybutyrate-co-3-hydroxyvalerate) (PHBV) are common choices for nanoparticle synthesis, but unlimited options are available [172]. Synthetic polymers can be easily tuned to exhibit desirable properties showing low toxicity, biocompatibility, and good drug encapsulation efficiency [173,174]. For example, poly (N-vinyl-2-pyrroildone) (PVP) is one of the promising polymers for use in colorectal cancer therapy [175]. On the one hand, the introduction of hydrophobic end groups allows the formation of aggregates of amphiphilic PVP chains capable of incorporating pharmacologically active substances, as well as selectively delivering them to the cell nucleus [176] without hydrolytic destruction in lysosomes. This opens up the potential for the delivery of labile oligonucleotides to cancer cells. On the other hand, PVP can be used to stabilize and functionalize metal nanoparticles. For example, PVP-functionalized palladium nanoparticles containing incorporated quercetin have shown significant inhibition of HCT-15 colorectal cancer cell proliferation [177]. 

PHBV/PLGA nanoparticles for 5-FU delivery were prepared by the double emulsion method. The obtained 5-FU polymeric drug-delivery platforms showed no hemolytic activity, as well as superior cytotoxicity and tumor growth inhibition effect compared to 5-FU [147]. PHBHV was also used as a single polymer for nanoparticle preparation, which showed excellent biocompatibility in its pristine form, but impacted HT-29 colorectal tumor cell metabolic status when loaded with 5-FU, presenting an intensified cytotoxic activity compared to free 5-FU [178]. Codelivery of 5-FU and OXP through PHBHV/PLGA nanocarriers augmented the cytotoxic and proapoptotic potential of free drugs and showed a promising tumor growth inhibition effect after administration in xenograft mouse cancer models [179]. 

Wu et al. [180] designed a complex multifunctional nanocarrier for delivery of both 5-FU and radionuclide iodine-131 (^131^I) by using a PEGylated PLA nanocarrier decorated with cetuximab (Cet-PEG-PLA-5-FU-^131^I). Using the solvent evaporation method, small stable spherical particles were generated that showed good entrapment of the 5-FU, which was preferentially released in acidic conditions. More, cetuximab functionalization triggers an enhancement of the cellular uptake of Cet-PEG-PLA-5-FU-^131^I by tumor cells in vitro and *in vivo,* where the Cet-PEG-PLA-5-FU-^131^I exhibited a superior antitumor effect compared with monotherapy. In another study, this research group targeted EGFR-overexpression of colorectal tumor cells by functionalizing PLGA nanocarriers with epidermal growth factor (EGF), followed by co-loading of 5-FU and perfluorocarbon (EGF-PLGA-5-FU-PFC). The use of perfluorocarbon as an artificial oxygen carrier to improve tumor oxygenation could act synergistically with 5-FU delivery, enhancing its cytotoxic effects. Indeed, perfluorocarbon presented an additive antitumor effect for 5-FU, as the most drastic in vivo antitumor effect was observed in the case of EGF-PLGA-5-FU-PFC administration [181]. 

Alginate was employed for assembly by the cross-linking process of HA-coated alginate nanogels functionalized with folic acid as encapsulation nanosystems of OXP (F/HA/AL/OXP). These systems inhibited HT-29 tumor cell viability in a time-dependent manner, based on the controlled release in time of OXP, with an enhanced cytotoxic effect as compared with free-OXP. The nanogels promoted apoptosis in HT-29 cell cultures better than free-OXP by modulating the expression of apoptosis key players Bax and Bcl-2 [182]. A hybrid polymer–silica nanosystem for OXP delivery was synthesized by Yang and colleagues [183] for the codelivery of tumor suppressor miRNA-204-5P and OXP through biocompatible HA-enriched PEI-mesoporous silica nanoparticles (Oxmi-HMSN). Following administration of Oxmi-HMSN, a synergistic anticancer effect was observed, the system showing promising efficiency in inducing cytotoxicity and apoptosis and in the diminution of tumor volume.

For IRI delivery, SN38 was conjugated with linoleic acid and encapsulated in poly (ethylene oxide)-poly (butylene oxide) (PEO-PBO) nanocarriers, which proved good stability and a controlled continuous release of the drug when compared with self-assembled linoleic acid-SN38 nanoparticles (SNPs). In vitro uptake studies revealed that the polymeric nanoshuttles are better internalized by HCT-116 colorectal tumor cells than SNPs, with an opposite uptake yield in case of macrophage treatment, which showed that the polymeric carrier could favor tumor cell uptake while reducing the risk for nanoparticle phagocytosis. Adding the increased stability in blood and enhanced tumor-targeting ability revealed by in vivo, it is clear that the PEO-PBO could ensure an increased concentration of SN38 at the tumor site than can be achieved by free-SN38 or SNP administration [184]. Salmanpour et al. [185] prepared poly(2-ethyl-2-oxazoline)-b-poly (L-glutamic acid) (PEtOx-b-PGlu) nanoparticles as carriers for SN38, chemically conjugated by carbodiimide mediated esterification. Grafting SN38 on these polymeric nanocarriers significantly increased SN38 cytotoxicity and determined an increased life span of murine CT26 models in comparison with free-IRI. 

Another class of polymeric nanoparticles is dendrimers, which present a unique radial architecture that consists of branches of polymers that originate from a central core, forming a tree-like structure [186]. Due to the highly branched organization, dendrimers can be easily functionalized on their outer surface to increase their biocompatibility or for active targeting purposes. For example, a combined strategy was used by Alibolandi et al. [187] that developed PEGylated poly(amidoamine) (PAMAM) dendrimers as carriers for camptothecin, which were further functionalized with AS1411 anti-nucleolin aptamers for achieving active-targeting nanosystems for CRC. The in vitro cytotoxicity results revealed that AS1411 functionalization can improve cellular uptake of the camptothecin nanocarrier as impacted severely on HT-29 and C26 colorectal tumor cell viability, which overexpress nucleolin receptors. More, to improve biocompatibility in blood, PEGylation has proven to be a smart strategy for avoiding hemolysis, which was significantly increased after administration of non-PEGylated dendrimers. In vivo results revealed that the PEG AS1411 PAMAM dendrimers loaded with camptothecin improved the free-drug pharmacokinetics and showed an enhanced effect for inhibiting C26 tumor growth, while protecting healthy tissue from alterations. 

### 4.3. Hybrid Drug-Delivery Systems 

As mentioned above, both lipid-based and polymer-based nanocarriers present their advantages and disadvantages. Based on the strengths of each nanosystem, lipid–polymer nanoparticles (LPNs) have emerged as hybrid drug-delivery systems that combine the two classes of materials into a hybrid nanocarrier with superior physicochemical and biological proprieties. By structure, LPNs are core–shell nanoparticles consisting of a polymeric core, where the drug cargo is encapsulated, protected by a lipid shell, covered by an optional hydrophilic polymeric stealth layer [188,189]. In this dual architecture, the high structural stability and controlled release of encapsulated drugs specific to polymeric nanoparticles are combined with the excellent biocompatibility of liposomes to design and develop next-generation core–shell nanoparticles. These LPNs show enhanced drug encapsulation efficiency and better control over the drug release profile, features that can be attributed to the protective lipid monolayer that seals the drug cargo into the polymeric core to avoid leakage [190,191]. For example, Wang et al. [121] successfully synthetized, by the solvent-evaporation method, HA-functionalized LPNs for codelivery of plasmid DNA and IRI, which showed good stability and enhanced cytotoxic effects on SW480 cells overexpressing CD44. More, in vivo results revealed that when loaded with plasmid DNA and IRI, HA-LPNs show an increased antitumor and transfection efficiency compared to non-functionalized LPNs, highlighting the remarkable potential of the HA-LPNs to act as targeted nanosystems for drug and gene combination therapy. 

## 5. Challenges in Drug-Delivery Systems Research

As highlighted in the previous section, numerous nanosized drug-delivery systems have been developed as drug carriers for CRC therapy, but most of the existing research is limited in presenting nanoparticle synthesis, characterization, and in vitro screening of antitumor performance. Despite obtaining promising results, the use of 2D colorectal tumor cell lines for drug-delivery system efficacy and mechanism of action investigation presents several disadvantages that highlight the need for better cancer models. For example, adherent epithelial tumor cells are grown as monolayers before treatment, an experimental setup that fails to mimic the complex 3D architecture of human solid tumors. As a result, all tumor cells are exposed to the provided treatment and receive oxygen and unlimited nutrients, aspects that fail to depict real tumor hallmarks such as an altered pattern of oxygen and nutrients delivery and the numerous biological barriers for nanoparticles to reach the tumor’s inner core [192]. More, the 2D cell cultures lack a tumor microenvironment and interactions with surrounding cells and the extracellular matrix, aspects that impact cell proliferative status, gene and protein expression, and responsiveness to applied treatments [193,194].

**Figure 4:**
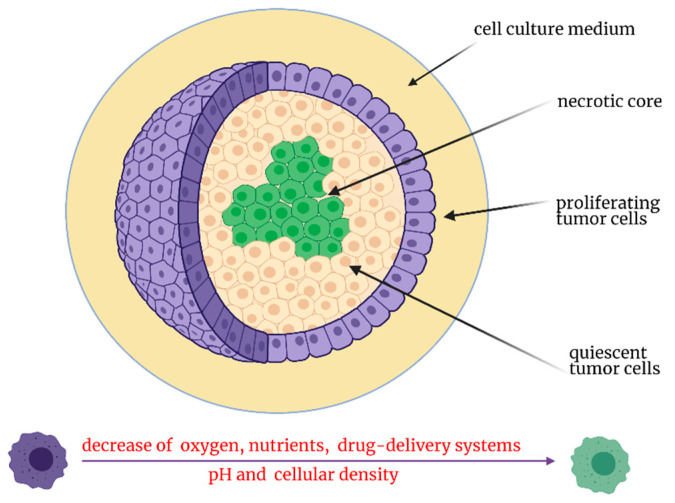
Schematic representation of a 3D multicellular tumor spheroid.

With the understanding of limitations imposed by traditional cell cultures and the need for a third dimension to recapitulate different features of epithelial tumors such as complex architecture and the gradient distribution of nutrients and treatments, researchers are starting to employ 3D tumor models for drug-delivery system screening. For this purpose, a popular choice is 3D tumor spheroids, which hold great promise in bridging the gap between in vitro and in vivo research by delivering biologically-relevant data about the screened nanosystems [195]. Besides for 3D tumor spheroids that can be generated by hanging drop, liquid overlay, spinner flasks, and microfluidic-based assembly, scaffold-based 3D cancer models are also available and require mechanical support that favors and sustains tumor cell organization into 3D structures [196]. Due to the 3D nature of spheroids, these cell culture models mimic several characteristics of real tumors that could be useful for a better and more realistic characterization of drug-delivery antitumor potential. First, despite the lack of vasculature and interaction with other cell types, 3D tumor spheroid architecture consists of multilayers of tumor cells organized in spherical compact aggregates. Therefore, the outer layers are represented by active proliferating cells, while the inner layers are represented by quiescent or senescent tumor cells [197]. Moreover, due to the gradient of oxygen and nutrients through the multicellular layers, 3D tumor spheroid hypoxia and enhanced lactate production affect the inner core of spheroids, aspects that lead to a decrease of pH, particulars that characterize human solid tumors [198]. Another strength of 3D tumor spheroids for drug-delivery system screening is the production of extracellular matrix that creates a physical barrier that limits the uniform distribution of drug-loaded nanoparticles within the 3D tumor cell mass [199,200]. The structure of 3D tumor spheroids is summarized in Figure 4. 

Based on the presented advantages, 3D tumor cell spheroids can be used for drug-delivery screening, and several nanosized systems for CRC have been tested in 3D models. For example, using ultra-low attachment culture plates, Smith et al. [201] generated 3D tumor spheroids by HCT116 colorectal adenocarcinoma cells for the screening of 5-FU loaded chitosan nanoparticle (5-FU CS NP) effects. The results highlighted the differences in response to 5-FU CS NP treatment between 2D and 3D cell cultures, where the same dose of 5-FU loaded CS NPs triggered a different effect on cell viability for HCT116 spheroids than HCT116 monolayers. An interesting study was carried out by Tchoryk et al. [202] who used the same cell line and generation method for obtaining 3D study models to investigate the impact of variation doxorubicin PGA nanoparticle physicochemical properties and compositions on nanoparticle penetration and uptake. Their results showed that size, PEGylation, and surface charge impact a nanocarrier’s potential for penetrating the 3D HCT116 spheroids. As 3D spheroids could be generated based on multiple cellular types, human intestinal fibroblasts and monocytes were combined with HCT116 tumor cells to generate a triple co-culture spheroid used for nanocarrier antitumor effect screening, which showed a lower yield in internalizing the studied nanoparticles than 2D monolayers [203]. 

However, despite the clear advantages of 3D cell cultures over 2D cell cultures, tumor cell monolayers still stand as the backbone of in vitro drug-delivery screening due to low costs and high reproducibility, as well as due to the perfect fit with the available laboratory techniques and methodologies, which need serious optimization to be adjusted for 3D cell models. At the current stage, 3D models are not capable of replacing animal testing, which can offer valuable information regarding nanoparticle tumor affinity, biodistribution, and excretion. As preclinical models, animal models still stand as the golden standard for assessing novel drugs or drug-delivery system effects, despite ethical issues and clear biological differences with humans [204,205]. However, the enhancement of 3D cancer cell models use for in vitro drug-delivery screening could represent an intermediary stage between 2D models and could diminish the overuse of animals for the screening of ineffective drug-delivery systems. The lack of an appropriate cancer model for in vitro studies, as well as the poor transition of satisfactory results from the preclinical stage to clinical trials, impact the difficulty that face drug-delivery systems to be approved by the Food and Drug Administration (FDA) and on the increasing number of unsuccessful clinical trials. Despite there are currently several clinical trials in advanced phases with drug-delivery systems developed for CRC [206], no nanosized systems have been approved until now by the FDA for CRC management. 

## 6. Conclusions and Future Outlook

This review aimed to cover the most recent advancements in organic nanosized drug-delivery systems for improving the efficacy and safety of current chemotherapy available for CRC, focusing on delivery systems designed for 5-FU, OXP, and IRI delivery. As an ideal drug-delivery system should be designed based on tumor biology and microenvironment-particular features, while considering the possible obstacle encountered until reaching the tumor site, it is no wonder that the latest developed nanoparticles are designed on an active targeting basis rather than classical drug-delivery systems, which have the advantage of the EPR effect to accumulate in tumors. For improving tumor selectivity, different singular or multiple approaches are employed for developing smart nanocarriers, with minimal biotoxicity for healthy cells, and with superior antitumor effects for malignant ones, by using nanoparticle surface functionalization or stimuli-responsive nanocarriers, as presented above. Since the chemotherapeutic agents administrated in CRC therapy are broad-spectrum anticancer agents used in the treatment of multiple malignancies, the organic nanosized drug-delivery systems could be screened for other types of cancer therapy. For example, 5-FU is a multifunctional anticancer agent administrated as therapy for multiple types of solid cancers such as breast cancer, gastric cancer, esophageal cancer, pancreatic cancer, glioblastoma, or melanoma [207]. Therefore, 5-FU loaded nanoparticles synthetized for CRC applications could be versatile and screened for various types of cancer applications. However, the synthesized parameters and formulation should be adjusted in dependence on the administration route (oral, intravenous, topical) and the biological barriers encountered after administration (skin barrier, blood–brain barrier, gastrointestinal tract, etc.) [208,209].

The main disadvantage to bridge the gap between preclinical research and clinics is the lack of appropriate cancer models for in vitro biological investigations. This issue slows the transition of the nanoparticles in clinics as a powerful tool for improving the current therapy available for CRC. In this view, approaching 3D strategies for developing in vitro colorectal cancer models could improve the odds for success for drug-delivery systems in clinical trials, while reducing animal use.

## Figures and Tables

**Table 1:** Examples of nanosized carriers as stimuli-responsive drug-delivery systems for CRC-targeted therapy.

Stimuli	Nanosystem Description	Biological Investigation Highlights	Ref.
Redox-responsive	Xylan-SS-curcumin nanoparticles loaded with 5-FU prodrug (5-FU-stearic acid)	Low hemolytic activity. Higher cytotoxicity than free drugs on HT-29 and HCT-115 cells.	[131]
pH-responsive	O’-methyl polyethylene glycol (omPEG) IRI liposomes and omPEG miR-200 solid lipid nanoparticle, both functionalized with mitochondria-targeting peptide K (RFKH)	Potent inductor of apoptosis that modulates effects of β-catenin/Multidrug Resistance (MDR)/apoptosis/Epithelial to Mesenchymal Transition (EMT) signaling pathways. In vivo superior tumor growth inhibition and low cytotoxicity on non-cancerous cells.	[132]
Enzyme-responsive	Doxorubicin c-RGD polytyrosine nanoparticles	Efficiently internalized by αvβ5 overexpressing HCT-116 colorectal cancer cells and highly cytotoxic. Improve survival rate of tumor-bearing mice by efficient tumor growth inhibition compared with free DOX or DOX liposomal formulation.	[133]
Magnetic-responsive	Hybrid liposome-magnetic nanoparticles loaded with Cy5.5 dye and oxaliplatin	Magnetic field stimulation enhanced cytotoxicity of nanoparticles in CC-531 adenocarcinoma cell cultures and directed the selective delivery of oxaliplatin at high concentrations in the targeted tissue.	[134]
Ultrasound-responsive	Anti-β-catenin small interfering RNA-loaded chitosan hydrochloride/carboxymethyl chitosan nanoparticle	Efficiently internalized by HT-29 tumor cells and successfully suppress in vitro expression of β-catenin.	[135]
Light-responsive	Polythiophene nanoparticles	Exert no cytotoxicity on colon carcinoma CT-26 cells in the range of 25–250 µg/mL concentration, while NIR laser-triggered photothermal treatment in nanoparticle pretreated CT-26 cell cultures triggers reduction of cell viability and apoptosis.	[136]

**Table 2:** Examples of various lipid compositions employed for liposome synthesis. Cationic lipids: DOTAP (1,2-dioleoyl-3-trimethylammoniun-propane); zwitterionic lipids: PC (phosphatidyl choline), DPPC (1,2-dihexadecanoyl-sn-glycero-3-phosphocholine); neutral lipids: DOPE (1,2-dioleoylsn-glycerol-3 phosphoethanolamine), CHOL (cholesterol); anionic lipids: DSPE (1, 2-distearoyl-sn-glycero-3-phosphoethanolamine-poly(ethylene glycol)).

Lipid Composition/Synthesis Method	Drug Cargo	Active Targeting	Ref.
DOTAP:DOPE:DSPE-PEG_2000_/thin layer film hydration method	5-FU	HA for CD44 receptor targeting	[118]
PC:CHOL:DSPE/thin layer film hydration method	5-FU	Transferrin for transferrin receptor (TFR) targeting	[100]
DPPC:CHOL:DSPE-PEG_2000_/thin layer film hydration method	5-FU	Folate for folate receptor (FR) targeting	[147]
PC:DSPE-PEG_2000_/ethanol injection method	rapamycin	Not applicable (NA)	[148]
PC-98T:DSPE-PEG_2000_:CHOL/thin layer film hydration method	SN38	HA	[149]
PC:DSPE-PEG_2000_/thin layer film hydration method	OXP	NA	[150]

## Data Availability

Data sharing not applicable.

## References

[1] Siegel R.L., Miller K.D., Jemal A. (2019). Cancer Statistics, 2019. Cancer J. Clin..

[2] Sung H., Ferlay J., Siegel R.L., Laversanne M., Soerjomataram I., Jemal A., Bray F. (2020). Global Cancer Statistics 2020: GLOBOCAN Estimates of Incidence and Mortality Worldwide for 36 Cancers in 185 Countries. Cancer J. Clin..

[3] Ferlizza E., Solmi R., Sgarzi M., Ricciardiello L., Lauriola M. (2021). The Roadmap of Colorectal Cancer Screening. Cancers.

[4] Fidler M.M., Soerjomataram I., Bray F. (2016). A Global View on Cancer Incidence and National Levels of the Human Development Index. Int. J. Cancer.

[5] Bray F., Ferlay J., Soerjomataram I., Siegel R.L., Torre L.A., Jemal A. (2018). Global Cancer Statistics 2018: GLOBOCAN Estimates of Incidence and Mortality Worldwide for 36 Cancers in 185 Countries. Cancer J. Clin..

[6] Thanikachalam K., Khan G. (2019). Colorectal Cancer and Nutrition. Nutrients.

[7] Bevan R., Rutter M.D. (2018). Colorectal Cancer Screening-Who, How, and When?. Clin. Endosc..

[8] Rawla P., Sunkara T., Barsouk A. (2019). Epidemiology of Colorectal Cancer: Incidence, Mortality, Survival, and Risk Factors. Przeglad Gastroenterol..

[9] Riihimäki M., Hemminki A., Sundquist J., Hemminki K. (2016). Patterns of Metastasis in Colon and Rectal Cancer. Sci. Rep..

[10] Vuik F.E., Nieuwenburg S.A., Bardou M., Lansdorp-Vogelaar I., Dinis-Ribeiro M., Bento M.J., Zadnik V., Pellisé M., Esteban L., Kaminski M.F. (2019). Increasing Incidence of Colorectal Cancer in Young Adults in Europe over the Last 25 Years. Gut.

[11] Wolf A.M., Fontham E.T., Church T.R., Flowers C.R., Guerra C.E., LaMonte S.J., Etzioni R., McKenna M.T., Oeffinger K.C., Shih Y.T. (2018). Colorectal Cancer Screening for Average-risk Adults: 2018 Guideline Update from the American Cancer Society. Cancer J. Clin..

[12] Nagtegaal I.D., Quirke P. (2008). What Is the Role for the Circumferential Margin in the Modern Treatment of Rectal Cancer?. J. Clin. Oncol..

[13] Heald R., Ryall R. (1986). Recurrence and Survival after Total Mesorectal Excision for Rectal Cancer. Lancet.

[14] Trastulli S., Cirocchi R., Listorti C., Cavaliere D., Avenia N., Gulla N., Giustozzi G., Sciannameo F., Noya G., Boselli C. (2012). Laparoscopic vs Open Resection for Rectal Cancer: A Meta-analysis of Randomized Clinical Trials. Colorectal Dis..

[15] Kapiteijn E., Marijnen C.A., Nagtegaal I.D., Putter H., Steup W.H., Wiggers T., Rutten H.J., Pahlman L., Glimelius B., van Krieken J.H.J. (2001). Preoperative Radiotherapy Combined with Total Mesorectal Excision for Resectable Rectal Cancer. N. Engl. J. Med..

[16] Bujko K., Nowacki M., Nasierowska-Guttmejer A., Michalski W., Bebenek M., Kryj M. (2006). Long-term Results of a Randomized Trial Comparing Preoperative Short-course Radiotherapy with Preoperative Conventionally Fractionated Chemoradiation for Rectal Cancer. Br. J. Surg..

[17] Kim J.H. (2015). Chemotherapy for Colorectal Cancer in the Elderly. World J. Gastroenterol..

[18] Gill S., Loprinzi C.L., Sargent D.J., Thomé S.D., Alberts S.R., Haller D.G., Benedetti J., Francini G., Shepherd L.E., Francois Seitz J. (2004). Pooled Analysis of Fluorouracil-Based Adjuvant Therapy for Stage II and III Colon Cancer: Who Benefits and by How Much?. J. Clin. Oncol..

[19] André T., Boni C., Navarro M., Tabernero J., Hickish T., Topham C., Bonetti A., Clingan P., Bridgewater J., Rivera F. (2009). Improved Overall Survival with Oxaliplatin, Fluorouracil, and Leucovorin as Adjuvant Treatment in Stage II or III Colon Cancer in the MOSAIC Trial. J. Clin. Oncol..

[20] Xie Y.-H., Chen Y.-X., Fang J.-Y. (2020). Comprehensive Review of Targeted Therapy for Colorectal Cancer. Signal Transduct. Target. Ther..

[21] Van Cutsem E., Cervantes A., Adam R., Sobrero A., Van Krieken J., Aderka D., Aguilar E.A., Bardelli A., Benson A., Bodoky G. (2016). ESMO Consensus Guidelines for the Management of Patients with Metastatic Colorectal Cancer. Ann. Oncol..

[22] Wang J., Li S., Liu Y., Zhang C., Li H., Lai B. (2020). Metastatic Patterns and Survival Outcomes in Patients with Stage IV Colon Cancer: A Population-based Analysis. Cancer Med..

[23] Blanco E., Shen H., Ferrari M. (2015). Principles of Nanoparticle Design for Overcoming Biological Barriers to Drug Delivery. Nat. Biotechnol..

[24] Kou L., Bhutia Y.D., Yao Q., He Z., Sun J., Ganapathy V. (2018). Transporter-Guided Delivery of Nanoparticles to Improve Drug Permeation across Cellular Barriers and Drug Exposure to Selective Cell Types. Front. Pharmacol..

[25] Gustavsson B., Carlsson G., Machover D., Petrelli N., Roth A., Schmoll H.-J., Tveit K.-M., Gibson F. (2015). A Review of the Evolution of Systemic Chemotherapy in the Management of Colorectal Cancer. Clin. Colorectal Cancer.

[26] Gill S., Thomas R., Goldberg R.M. (2003). Colorectal Cancer Chemotherapy. Aliment. Pharmacol. Ther..

[27] Alcindor T., Beauger N. (2011). Oxaliplatin: A Review in the Era of Molecularly Targeted Therapy. Curr. Oncol..

[28] Zhang N., Yin Y., Xu S.-J., Chen W.-S. (2008). 5-Fluorouracil: Mechanisms of Resistance and Reversal Strategies. Molecules.

[29] Fujita K., Kubota Y., Ishida H., Sasaki Y. (2015). Irinotecan, a Key Chemotherapeutic Drug for Metastatic Colorectal Cancer. World J. Gastroenterol..

[30] Diasio R.B., Harris B.E. (1989). Clinical Pharmacology of 5-Fluorouracil. Clin. Pharmacokinet..

[31] Kümler I., Sørensen P.G., Palshof J., Høgdall E., Skovrider-Ruminski W., Theile S., Fullerton A., Nielsen P., Jensen B.V., Nielsen D. (2019). Oral Administration of Irinotecan in Patients with Solid Tumors: An Open-Label, Phase I, Dose Escalating Study Evaluating Safety, Tolerability and Pharmacokinetics. Cancer Chemother. Pharmacol..

[32] Hsu H., Chen M., Baskaran R., Lin Y., Day C.H., Lin Y., Tu C., Vijaya Padma V., Kuo W., Huang C. (2018). Oxaliplatin Resistance in Colorectal Cancer Cells Is Mediated via Activation of ABCG2 to Alleviate ER Stress Induced Apoptosis. J. Cell. Physiol..

[33] Xu Y., Villalona-Calero M. (2002). Irinotecan: Mechanisms of Tumor Resistance and Novel Strategies for Modulating Its Activity. Ann. Oncol..

[34] Negrei C., Hudita A., Ginghina O., Galateanu B., Voicu S.N., Stan M., Costache M., Fenga C., Drakoulis N., Tsatsakis A.M. (2016). Colon Cancer Cells Gene Expression Signature as Response to 5-Fluorouracil, Oxaliplatin, and Folinic Acid Treatment. Front. Pharmacol..

[35] Johnson M.R., Hageboutros A., Wang K., High L., Smith J.B., Diasio R.B. (1999). Life-Threatening Toxicity in a Dihydropyrimidine Dehydrogenase-Deficient Patient after Treatment with Topical 5-Fluorouracil. Clin. Cancer Res..

[36] Nikolouzakis T.K., Stivaktakis P.D., Apalaki P., Kalliantasi K., Sapsakos T.M., Spandidos D.A., Tsatsakis A., Souglakos J., Tsiaoussis J. (2019). Effect of Systemic Treatment on the Micronuclei Frequency in the Peripheral Blood of Patients with Metastatic Colorectal Cancer. Oncol. Lett..

[37] Oun R., Moussa Y.E., Wheate N.J. (2018). The Side Effects of Platinum-Based Chemotherapy Drugs: A Review for Chemists. Dalton Trans..

[38] Braun M.S., Seymour M.T. (2011). Balancing the Efficacy and Toxicity of Chemotherapy in Colorectal Cancer. Ther. Adv. Med. Oncol..

[39] Tariman J.D. (2017). Changes in Cancer Treatment: Mabs, Mibs, Mids, Nabs, and Nibs. Nurs. Clin..

[40] Cremolini C., Loupakis F., Antoniotti C., Lupi C., Sensi E., Lonardi S., Mezi S., Tomasello G., Ronzoni M., Zaniboni A. (2015). FOLFOXIRI plus Bevacizumab versus FOLFIRI plus Bevacizumab as First-Line Treatment of Patients with Metastatic Colorectal Cancer: Updated Overall Survival and Molecular Subgroup Analyses of the Open-Label, Phase 3 TRIBE Study. Lancet Oncol..

[41] Cunningham D., Lang I., Marcuello E., Lorusso V., Ocvirk J., Shin D.B., Jonker D., Osborne S., Andre N., Waterkamp D. (2013). Bevacizumab plus Capecitabine versus Capecitabine Alone in Elderly Patients with Previously Untreated Metastatic Colorectal Cancer (AVEX): An Open-Label, Randomised Phase 3 Trial. Lancet Oncol..

[42] Simkens L.H., van Tinteren H., May A., ten Tije A.J., Creemers G.-J.M., Loosveld O.J., de Jongh F.E., Erdkamp F.L., Erjavec Z., van der Torren A.M. (2015). Maintenance Treatment with Capecitabine and Bevacizumab in Metastatic Colorectal Cancer (CAIRO3): A Phase 3 Randomised Controlled Trial of the Dutch Colorectal Cancer Group. Lancet.

[43] Tabernero J., Yoshino T., Cohn A.L., Obermannova R., Bodoky G., Garcia-Carbonero R., Ciuleanu T.-E., Portnoy D.C., Van Cutsem E., Grothey A. (2015). Ramucirumab versus Placebo in Combination with Second-Line FOLFIRI in Patients with Metastatic Colorectal Carcinoma That Progressed during or after First-Line Therapy with Bevacizumab, Oxaliplatin, and a Fluoropyrimidine (RAISE): A Randomised, Double-Blind, Multicentre, Phase 3 Study. Lancet Oncol..

[44] Folprecht G., Pericay C., Saunders M.P., Thomas A., Lopez R.L., Roh J., Chistyakov V., Höhler T., Kim J.-S., Hofheinz R.-D. (2016). Oxaliplatin and 5-FU/Folinic Acid (Modified FOLFOX6) with or without Aflibercept in First-Line Treatment of Patients with Metastatic Colorectal Cancer: The AFFIRM Study. Ann. Oncol..

[45] Van Cutsem E., Tabernero J., Lakomy R., Prenen H., Prausová J., Macarulla T., Ruff P., Van Hazel G.A., Moiseyenko V., Ferry D. (2012). Addition of Aflibercept to Fluorouracil, Leucovorin, and Irinotecan Improves Survival in a Phase III Randomized Trial in Patients with Metastatic Colorectal Cancer Previously Treated with an Oxaliplatin-Based Regimen. J. Clin. Oncol..

[46] Zuazo-Gaztelu I., Casanovas O. (2018). Unraveling the Role of Angiogenesis in Cancer Ecosystems. Front. Oncol..

[47] Folkman J. (2002). Role of Angiogenesis in Tumor Growth and Metastasis.

[48] Seymour M.T., Brown S.R., Middleton G., Maughan T., Richman S., Gwyther S., Lowe C., Seligmann J.F., Wadsley J., Maisey N. (2013). Panitumumab and Irinotecan versus Irinotecan Alone for Patients with KRAS Wild-Type, Fluorouracil-Resistant Advanced Colorectal Cancer (PICCOLO): A Prospectively Stratified Randomised Trial. Lancet Oncol..

[49] Douillard J.-Y., Siena S., Cassidy J., Tabernero J., Burkes R., Barugel M., Humblet Y., Bodoky G., Cunningham D., Jassem J. (2010). Randomized, Phase III Trial of Panitumumab with Infusional Fluorouracil, Leucovorin, and Oxaliplatin (FOLFOX4) versus FOLFOX4 Alone as First-Line Treatment in Patients with Previously Untreated Metastatic Colorectal Cancer: The PRIME Study. J. Clin. Oncol..

[50] Van Cutsem E., Köhne C.-H., Hitre E., Zaluski J., Chang Chien C.-R., Makhson A., D’Haens G., Pintér T., Lim R., Bodoky G. (2009). Cetuximab and Chemotherapy as Initial Treatment for Metastatic Colorectal Cancer. N. Engl. J. Med..

[51] Markman B., Javier Ramos F., Capdevila J., Tabernero J. (2010). EGFR and KRAS in Colorectal Cancer. Adv. Clin. Chem..

[52] Wee P., Wang Z. (2017). Epidermal Growth Factor Receptor Cell Proliferation Signaling Pathways. Cancers.

[53] Roskoski R. (2019). Small Molecule Inhibitors Targeting the EGFR/ErbB Family of Protein-Tyrosine Kinases in Human Cancers. Pharmacol. Res..

[54] Wang Z. (2017). ErbB Receptors and Cancer. ErbB Recept. Signal..

[55] Hubbard J.M., Grothey A. (2015). Progress in Defining First-Line and Maintenance Therapies. Nat. Rev. Clin. Oncol..

[56] Loupakis F., Cremolini C., Salvatore L., Masi G., Sensi E., Schirripa M., Michelucci A., Pfanner E., Brunetti I., Lupi C. (2014). FOLFOXIRI plus Bevacizumab as First-Line Treatment in BRAF Mutant Metastatic Colorectal Cancer. Eur. J. Cancer.

[57] Zhang J., Tang H., Liu Z., Chen B. (2017). Effects of Major Parameters of Nanoparticles on Their Physical and Chemical Properties and Recent Application of Nanodrug Delivery System in Targeted Chemotherapy. Int. J. Nanomed..

[58] Pittella F., Zhang M., Lee Y., Kim H.J., Tockary T., Osada K., Ishii T., Miyata K., Nishiyama N., Kataoka K. (2011). Enhanced Endosomal Escape of SiRNA-Incorporating Hybrid Nanoparticles from Calcium Phosphate and PEG-Block Charge-Conversional Polymer for Efficient Gene Knockdown with Negligible Cytotoxicity. Biomaterials.

[59] Graf C., Gao Q., Schütz I., Noufele C.N., Ruan W., Posselt U., Korotianskiy E., Nordmeyer D., Rancan F., Hadam S. (2012). Surface Functionalization of Silica Nanoparticles Supports Colloidal Stability in Physiological Media and Facilitates Internalization in Cells. Langmuir.

[60] Tang J., Li L., Howard C.B., Mahler S.M., Huang L., Xu Z.P. (2015). Preparation of Optimized Lipid-Coated Calcium Phosphate Nanoparticles for Enhanced in vitro Gene Delivery to Breast Cancer Cells. J. Mater. Chem. B.

[61] Limbach L.K., Li Y., Grass R.N., Brunner T.J., Hintermann M.A., Muller M., Gunther D., Stark W.J. (2005). Oxide Nanoparticle Uptake in Human Lung Fibroblasts:  Effects of Particle Size, Agglomeration, and Diffusion at Low Concentrations. Environ. Sci. Technol..

[62] Herd H., Daum N., Jones A.T., Huwer H., Ghandehari H., Lehr C.-M. (2013). Nanoparticle Geometry and Surface Orientation Influence Mode of Cellular Uptake. ACS Nano.

[63] Sabourian P., Yazdani G., Ashraf S.S., Frounchi M., Mashayekhan S., Kiani S., Kakkar A. (2020). Effect of Physico-Chemical Properties of Nanoparticles on Their Intracellular Uptake. Int. J. Mol. Sci..

[64] Liu Y., Tan J., Thomas A., Ou-Yang D., Muzykantov V.R. (2012). The Shape of Things to Come: Importance of Design in Nanotechnology for Drug Delivery. Ther. Deliv..

[65] Iftode A., Drăghici G.A., Macașoi I., Marcovici I., Coricovac D.E., Dragoi R., Tischer A., Kovatsi L., Tsatsakis A.M., Cretu O. (2021). Exposure to Cadmium and Copper Triggers Cytotoxic Effects and Epigenetic Changes in Human Colorectal Carcinoma HT-29 Cells. Exp. Ther. Med..

[66] Krasanakis T., Nikolouzakis T.K., Sgantzos M., Mariolis-Sapsakos T., Souglakos J., Spandidos D.A., Tsitsimpikou C., Tsatsakis A., Tsiaoussis J. (2019). Role of Anabolic Agents in Colorectal Carcinogenesis: Myths and Realities (Review). Oncol. Rep..

[67] Ashraf N., Mahipal A., Kim R. (2013). Viral Vector Vaccines to Treat Colorectal Cancer. Curr. Colorectal Cancer Rep..

[68] Cheng X.J., Lin J.C., Ding Y.F., Zhu L., Ye J., Tu S.P. (2016). Survivin Inhibitor YM155 Suppresses Gastric Cancer Xenograft Growth in Mice without Affecting Normal Tissues. Oncotarget.

[69] Basak S., Eck S., Gutzmer R., Smith A.J., Birebent B., Purev E., Staib L., Somasundaram R., Zaloudik J., Li W. (2000). Colorectal Cancer Vaccines: Antiidiotypic Antibody, Recombinant Protein, and Viral Vector. Ann. N. Y. Acad. Sci..

[70] Farnsworth R.H., Lackmann M., Achen M.G., Stacker S.A. (2014). Vascular Remodeling in Cancer. Oncogene.

[71] Cao Y. (2009). Tumor Angiogenesis and Molecular Targets for Therapy. Front. Biosci. Landmark Ed..

[72] Carmeliet P., Jain R.K. (2000). Angiogenesis in Cancer and Other Diseases. Nature.

[73] Siemann D.W. (2011). The Unique Characteristics of Tumor Vasculature and Preclinical Evidence for Its Selective Disruption by Tumor-Vascular Disrupting Agents. Cancer Treat. Rev..

[74] Gee M.S., Procopio W.N., Makonnen S., Feldman M.D., Yeilding N.M., Lee W.M. (2003). Tumor Vessel Development and Maturation Impose Limits on the Effectiveness of Anti-Vascular Therapy. Am. J. Pathol..

[75] McDonald D.M., Baluk P. (2002). Significance of Blood Vessel Leakiness in Cancer. Cancers Res..

[76] Hashizume H., Baluk P., Morikawa S., McLean J.W., Thurston G., Roberge S., Jain R.K., McDonald D.M. (2000). Openings between Defective Endothelial Cells Explain Tumor Vessel Leakiness. Am. J. Pathol..

[77] Kalyane D., Raval N., Maheshwari R., Tambe V., Kalia K., Tekade R.K. (2019). Employment of Enhanced Permeability and Retention Effect (EPR): Nanoparticle-Based Precision Tools for Targeting of Therapeutic and Diagnostic Agent in Cancer. Mater. Sci. Eng. C.

[78] Yuan F., Dellian M., Fukumura D., Leunig M., Berk D.A., Torchilin V.P., Jain R.K. (1995). Vascular Permeability in a Human Tumor Xenograft: Molecular Size Dependence and Cutoff Size. Cancer Res..

[79] Hobbs S.K., Monsky W.L., Yuan F., Roberts W.G., Griffith L., Torchilin V.P., Jain R.K. (1998). Regulation of Transport Pathways in Tumor Vessels: Role of Tumor Type and Microenvironment. Proc. Natl. Acad. Sci. USA.

[80] Chen Y., Lo C., Hsiue G. (2014). Multifunctional Nanomicellar Systems for Delivering Anticancer Drugs. J. Biomed. Mater. Res. A.

[81] Fang J., Nakamura H., Maeda H. (2011). The EPR Effect: Unique Features of Tumor Blood Vessels for Drug Delivery, Factors Involved, and Limitations and Augmentation of the Effect. Adv. Drug Deliv. Rev..

[82] Alexis F., Pridgen E., Molnar L.K., Farokhzad O.C. (2008). Factors Affecting the Clearance and Biodistribution of Polymeric Nanoparticles. Mol. Pharm..

[83] Jokerst J.V., Lobovkina T., Zare R.N., Gambhir S.S. (2011). Nanoparticle PEGylation for Imaging and Therapy. Nanomedicine.

[84] Gabizon A.A., Shmeeda H., Zalipsky S. (2006). Pros and Cons of the Liposome Platform in Cancer Drug Targeting. J. Liposome Res..

[85] Gulati N.M., Stewart P.L., Steinmetz N.F. (2018). Bioinspired Shielding Strategies for Nanoparticle Drug Delivery Applications. Mol. Pharm..

[86] Turecek P.L., Bossard M.J., Schoetens F., Ivens I.A. (2016). PEGylation of Biopharmaceuticals: A Review of Chemistry and Nonclinical Safety Information of Approved Drugs. J. Pharm. Sci..

[87] Immordino M.L., Dosio F., Cattel L. (2006). Stealth Liposomes: Review of the Basic Science, Rationale, and Clinical Applications, Existing and Potential. Int. J. Nanomed..

[88] D’souza A.A., Shegokar R. (2016). Polyethylene Glycol (PEG): A Versatile Polymer for Pharmaceutical Applications. Expert Opin. Drug Deliv..

[89] Knop K., Hoogenboom R., Fischer D. (2010). Schubert, U.S. Poly (Ethylene Glycol) in Drug Delivery: Pros and Cons as Well as Potential Alternatives. Angew. Chem. Int. Ed..

[90] Fang Y., Xue J., Gao S., Lu A., Yang D., Jiang H., He Y., Shi K. (2017). Cleavable PEGylation: A Strategy for Overcoming the “PEG Dilemma” in Efficient Drug Delivery. Drug Deliv..

[91] Hatakeyama H., Akita H., Harashima H. (2013). The Polyethyleneglycol Dilemma: Advantage and Disadvantage of PEGylation of Liposomes for Systemic Genes and Nucleic Acids Delivery to Tumors. Biol. Pharm. Bull..

[92] Daniels T.R., Delgado T., Rodriguez J.A., Helguera G., Penichet M.L. (2006). The Transferrin Receptor Part I: Biology and Targeting with Cytotoxic Antibodies for the Treatment of Cancer. Clin. Immunol..

[93] Lopez A., Cacoub P., Macdougall I.C., Peyrin-Biroulet L. (2016). Iron Deficiency Anaemia. Lancet.

[94] Prutki M., Poljak-Blazi M., Jakopovic M., Tomas D., Stipancic I., Zarkovic N. (2006). Altered Iron Metabolism, Transferrin Receptor 1 and Ferritin in Patients with Colon Cancer. Cancer Lett..

[95] Xue X., Shah Y.M. (2013). Intestinal Iron Homeostasis and Colon Tumorigenesis. Nutrients.

[96] Okazaki F., Matsunaga N., Okazaki H., Utoguchi N., Suzuki R., Maruyama K., Koyanagi S., Ohdo S. (2010). Circadian Rhythm of Transferrin Receptor 1 Gene Expression Controlled by C-Myc in Colon Cancer–Bearing Mice. Cancer Res..

[97] Sardoiwala M.N., Kushwaha A.C., Dev A., Shrimali N., Guchhait P., Karmakar S., Roy Choudhury S. (2020). Hypericin-Loaded Transferrin Nanoparticles Induce PP2A-Regulated BMI1 Degradation in Colorectal Cancer-Specific Chemo-Photodynamic Therapy. ACS Biomater. Sci. Eng..

[98] Wei Y., Gu X., Sun Y., Meng F., Storm G., Zhong Z. (2020). Transferrin-Binding Peptide Functionalized Polymersomes Mediate Targeted Doxorubicin Delivery to Colorectal Cancer in Vivo. J. Control. Release.

[99] Ahmed F., Kumari S., Kondapi A.K. (2018). Evaluation of Antiproliferative Activity, Safety and Biodistribution of Oxaliplatin and 5-Fluorouracil Loaded Lactoferrin Nanoparticles for the Management of Colon Adenocarcinoma: An in vitro and an in Vivo Study. Pharm. Res..

[100] Moghimipour E., Rezaei M., Ramezani Z., Kouchak M., Amini M., Angali K.A., Dorkoosh F.A., Handali S. (2018). Transferrin Targeted Liposomal 5-Fluorouracil Induced Apoptosis via Mitochondria Signaling Pathway in Cancer Cells. Life Sci..

[101] Quici S., Casoni A., Foschi F., Armelao L., Bottaro G., Seraglia R., Bolzati C., Salvarese N., Carpanese D., Rosato A. (2015). Folic Acid-Conjugated Europium Complexes as Luminescent Probes for Selective Targeting of Cancer Cells. J. Med. Chem..

[102] Fernández M., Javaid F., Chudasama V. (2018). Advances in Targeting the Folate Receptor in the Treatment/Imaging of Cancers. Chem. Sci..

[103] Tiernan J., Perry S., Verghese E., West N., Yeluri S., Jayne D., Hughes T. (2013). Carcinoembryonic Antigen Is the Preferred Biomarker for in Vivo Colorectal Cancer Targeting. Br. J. Cancer.

[104] Chen Y.-L., Chang M.-C., Huang C.-Y., Chiang Y.-C., Lin H.-W., Chen C.-A., Hsieh C.-Y., Cheng W.-F. (2012). Serous Ovarian Carcinoma Patients with High Alpha-Folate Receptor Had Reducing Survival and Cytotoxic Chemo-Response. Mol. Oncol..

[105] Süntar I., Yakıncı Ö.F. (2020). Potential risks of phytonutrients associated with high-dose or long-term use. Phytonutrients in Food.

[106] Vlahov I.R., Leamon C.P. (2012). Engineering Folate–Drug Conjugates to Target Cancer: From Chemistry to Clinic. Bioconjug. Chem..

[107] Ledermann J., Canevari S., Thigpen T. (2015). Targeting the Folate Receptor: Diagnostic and Therapeutic Approaches to Personalize Cancer Treatments. Ann. Oncol..

[108] Ruman U., Buskaran K., Pastorin G., Masarudin M.J., Fakurazi S., Hussein M.Z. (2021). Synthesis and Characterization of Chitosan-Based Nanodelivery Systems to Enhance the Anticancer Effect of Sorafenib Drug in Hepatocellular Carcinoma and Colorectal Adenocarcinoma Cells. Nanomaterials.

[109] de Oliveira A.L.C., Zerillo L., Cruz L.J., Schomann T., Chan A.B., de Carvalho T.G., Souza S.V., de Araújo A.A., de Geus-Oei L.-F., de Araújo Júnior R.F. (2021). Maximizing the Potency of Oxaliplatin Coated Nanoparticles with Folic Acid for Modulating Tumor Progression in Colorectal Cancer. Mater. Sci. Eng. C.

[110] Martín M.J., Azcona P., Lassalle V., Gentili C. (2021). Doxorubicin Delivery by Magnetic Nanotheranostics Enhances the Cell Death in Chemoresistant Colorectal Cancer-Derived Cells. Eur. J. Pharm. Sci..

[111] Soe Z.C., Poudel B.K., Nguyen H.T., Thapa R.K., Ou W., Gautam M., Poudel K., Jin S.G., Jeong J.-H., Ku S.K. (2019). Folate-Targeted Nanostructured Chitosan/Chondroitin Sulfate Complex Carriers for Enhanced Delivery of Bortezomib to Colorectal Cancer Cells. Asian J. Pharm. Sci..

[112] Rajpoot K., Jain S.K. (2020). Oral Delivery of PH-Responsive Alginate Microbeads Incorporating Folic Acid-Grafted Solid Lipid Nanoparticles Exhibits Enhanced Targeting Effect against Colorectal Cancer: A Dual-Targeted Approach. Int. J. Biol. Macromol..

[113] Ansari L., Derakhshi M., Bagheri E., Shahtahmassebi N., Malaekeh-Nikouei B. (2020). Folate Conjugation Improved Uptake and Targeting of Porous Hydroxyapatite Nanoparticles Containing Epirubicin to Cancer Cells. Pharm. Dev. Technol..

[114] Kumar C.S., Thangam R., Mary S.A., Kannan P.R., Arun G., Madhan B. (2020). Targeted Delivery and Apoptosis Induction of Trans-Resveratrol-Ferulic Acid Loaded Chitosan Coated Folic Acid Conjugate Solid Lipid Nanoparticles in Colon Cancer Cells. Carbohydr. Polym..

[115] Wang Z., Tang Y., Xie L., Huang A., Xue C., Gu Z., Wang K., Zong S. (2019). The Prognostic and Clinical Value of CD44 in Colorectal Cancer: A Meta-Analysis. Front. Oncol..

[116] Du L., Wang H., He L., Zhang J., Ni B., Wang X., Jin H., Cahuzac N., Mehrpour M., Lu Y. (2008). CD44 Is of Functional Importance for Colorectal Cancer Stem Cells. Clin. Cancer Res..

[117] Jing F., Kim H.J., Kim C.H., Kim Y.J., Lee J.H., Kim H.R. (2015). Colon Cancer Stem Cell Markers CD44 and CD133 in Patients with Colorectal Cancer and Synchronous Hepatic Metastases. Int. J. Oncol..

[118] Mansoori B., Mohammadi A., Abedi-Gaballu F., Abbaspour S., Ghasabi M., Yekta R., Shirjang S., Dehghan G., Hamblin M.R., Baradaran B. (2020). Hyaluronic Acid-decorated Liposomal Nanoparticles for Targeted Delivery of 5-fluorouracil into HT-29 Colorectal Cancer Cells. J. Cell. Physiol..

[119] Pan D.C., Krishnan V., Salinas A.K., Kim J., Sun T., Ravid S., Peng K., Wu D., Nurunnabi M., Nelson J.A. (2021). Hyaluronic a Cid–Doxorubicin Nanoparticles for Targeted Treatment of Colorectal Cancer. Bioeng. Transl. Med..

[120] Qu C.-Y., Zhou M., Chen Y., Chen M., Shen F., Xu L.-M. (2015). Engineering of Lipid Prodrug-Based, Hyaluronic Acid-Decorated Nanostructured Lipid Carriers Platform for 5-Fluorouracil and Cisplatin Combination Gastric Cancer Therapy. Int. J. Nanomed..

[121] Wang Z., Zang A., Wei Y., An L., Hong D., Shi Y., Zhang J., Su S., Fang G. (2020). Hyaluronic Acid Capped, Irinotecan and Gene Co-Loaded Lipid-Polymer Hybrid Nanocarrier-Based Combination Therapy Platform for Colorectal Cancer. Drug Des. Devel. Ther..

[122] Zhang X., Zhao M., Cao N., Qin W., Zhao M., Wu J., Lin D. (2020). Construction of a Tumor Microenvironment PH-Responsive Cleavable PEGylated Hyaluronic Acid Nano-Drug Delivery System for Colorectal Cancer Treatment. Biomater. Sci..

[123] Liszbinski R.B., Romagnoli G.G., Gorgulho C.M., Basso C.R., Pedrosa V.A., Kaneno R. (2020). Anti-EGFR-Coated Gold Nanoparticles in vitro Carry 5-Fluorouracil to Colorectal Cancer Cells. Materials.

[124] Bhattacharya S. (2020). Anti-EGFR-MAb and 5-Fluorouracil Conjugated Polymeric Nanoparticle for Colorectal Cancer. Recent Pat. Anticancer Drug Discov..

[125] Chen R., Huang Y., Wang L., Zhou J., Tan Y., Peng C., Yang P., Peng W., Li J., Gu Q. (2021). Cetuximab Functionalization Strategy for Combining Active Targeting and Antimigration Capacities of a Hybrid Composite Nanoplatform Applied to Deliver 5-Fluorouracil: Toward Colorectal Cancer Treatment. Biomater. Sci..

[126] Mokhtarzadeh A., Tabarzad M., Ranjbari J., de la Guardia M., Hejazi M., Ramezani M. (2016). Aptamers as Smart Ligands for Nano-Carriers Targeting. TrAC Trends Anal. Chem..

[127] Yao F., An Y., Li X., Li Z., Duan J., Yang X.-D. (2020). Targeted Therapy of Colon Cancer by Aptamer-Guided Holliday Junctions Loaded with Doxorubicin. Int. J. Nanomed..

[128] Thomas R.G., Surendran S.P., Jeong Y.Y. (2020). Tumor Microenvironment-Stimuli Responsive Nanoparticles for Anticancer Therapy. Front. Mol. Biosci..

[129] Vaupel P., Kallinowski F., Okunieff P. (1989). Blood Flow, Oxygen and Nutrient Supply, and Metabolic Microenvironment of Human Tumors: A Review. Cancer Res..

[130] Yu L., Chen X., Sun X., Wang L., Chen S. (2017). The Glycolytic Switch in Tumors: How Many Players Are Involved?. J. Cancer.

[131] Kumar B., Priyadarshi R., Deeba F., Kulshreshtha A., Kumar A., Agrawal G., Gopinath P., Negi Y.S. (2020). Redox Responsive Xylan-SS-Curcumin Prodrug Nanoparticles for Dual Drug Delivery in Cancer Therapy. Mater. Sci. Eng. C.

[132] Juang V., Chang C., Wang C., Wang H., Lo Y. (2019). PH-Responsive PEG-Shedding and Targeting Peptide-Modified Nanoparticles for Dual-Delivery of Irinotecan and MicroRNA to Enhance Tumor-Specific Therapy. Small.

[133] Gu X., Wei Y., Fan Q., Sun H., Cheng R., Zhong Z., Deng C. (2019). CRGD-Decorated Biodegradable Polytyrosine Nanoparticles for Robust Encapsulation and Targeted Delivery of Doxorubicin to Colorectal Cancer in Vivo. J. Control. Release.

[134] Gogineni V.R., Maddirela D.R., Park W., Jagtap J.M., Parchur A.K., Sharma G., Ibrahim E.-S., Joshi A., Larson A.C., Kim D.-H. (2020). Localized and Triggered Release of Oxaliplatin for the Treatment of Colorectal Liver Metastasis. J. Cancer.

[135] Yan L., Gao S., Shui S., Liu S., Qu H., Liu C., Zheng L. (2020). Small Interfering RNA-Loaded Chitosan Hydrochloride/Carboxymethyl Chitosan Nanoparticles for Ultrasound-Triggered Release to Hamper Colorectal Cancer Growth in Vitro. Int. J. Biol. Macromol..

[136] Bhattarai D.P., Kim B.S. (2020). NIR-Triggered Hyperthermal Effect of Polythiophene Nanoparticles Synthesized by Surfactant-Free Oxidative Polymerization Method on Colorectal Carcinoma Cells. Cells.

[137] Maiyo F., Singh M. (2017). Selenium Nanoparticles: Potential in Cancer Gene and Drug Delivery. Nanomedicine.

[138] Abedini F., Hosseinkhani H., Ismail M., Domb A.J., Omar A.R., Chong P.P., Hong P.-D., Yu D.-S., Farber I.-Y. (2012). Cationized Dextran Nanoparticle-Encapsulated CXCR4-SiRNA Enhanced Correlation between CXCR4 Expression and Serum Alkaline Phosphatase in a Mouse Model of Colorectal Cancer. Int. J. Nanomed..

[139] Marquez J., Fernandez-Piñeiro I., Araúzo-Bravo M.J., Poschmann G., Stühler K., Khatib A., Sanchez A., Unda F., Ibarretxe G., Bernales I. (2018). Targeting Liver Sinusoidal Endothelial Cells with Mi R-20a-loaded Nanoparticles Reduces Murine Colon Cancer Metastasis to the Liver. Int. J. Cancer.

[140] Bangham A., Standish M.M., Watkins J.C. (1965). Diffusion of Univalent Ions across the Lamellae of Swollen Phospholipids. J. Mol. Biol..

[141] Bulbake U., Doppalapudi S., Kommineni N., Khan W. (2017). Liposomal formulations in clinical use: An updated review. Pharmaceutics.

[142] Lamichhane N., Udayakumar T.S., D’Souza W.D., Simone C.B., Raghavan S.R., Polf J., Mahmood J. (2018). Liposomes: Clinical Applications and Potential for Image-Guided Drug Delivery. Molecules.

[143] Alavi M., Karimi N., Safaei M. (2017). Application of Various Types of Liposomes in Drug Delivery Systems. Adv. Pharm. Bull..

[144] Gonda A., Zhao N., Shah J.V., Calvelli H.R., Kantamneni H., Francis N.L., Ganapathy V. (2019). Engineering Tumor-Targeting Nanoparticles as Vehicles for Precision Nanomedicine. Med. One.

[145] Akbarzadeh A., Rezaei-Sadabady R., Davaran S., Joo S.W., Zarghami N., Hanifehpour Y., Samiei M., Kouhi M., Nejati-Koshki K. (2013). Liposome: Classification, Preparation, and Applications. Nanoscale Res. Lett..

[146] Li M., Du C., Guo N., Teng Y., Meng X., Sun H., Li S., Yu P., Galons H. (2019). Composition Design and Medical Application of Liposomes. Eur. J. Med. Chem..

[147] Handali S., Moghimipour E., Rezaei M., Ramezani Z., Kouchak M., Amini M., Angali K.A., Saremy S., Dorkoosh F.A. (2018). A Novel 5-Fluorouracil Targeted Delivery to Colon Cancer Using Folic Acid Conjugated Liposomes. Biomed. Pharmacother..

[148] Chen Y.-Q., Zhu W.-T., Lin C.-Y., Yuan Z.-W., Li Z.-H., Yan P.-K. (2021). Delivery of Rapamycin by Liposomes Synergistically Enhances the Chemotherapy Effect of 5-Fluorouracil on Colorectal Cancer. Int. J. Nanomed..

[149] Wu C., Zhang Y., Yang D., Zhang J., Ma J., Cheng D., Chen J., Deng L. (2019). Novel SN38 Derivative-Based Liposome as Anticancer Prodrug: An in vitro and in Vivo Study. Int. J. Nanomed..

[150] Garcia-Pinel B., Jabalera Y., Ortiz R., Cabeza L., Jimenez-Lopez C., Melguizo C., Prados J. (2020). Biomimetic Magnetoliposomes as Oxaliplatin Nanocarriers: In vitro Study for Potential Application in Colon Cancer. Pharmaceutics.

[151] Zhang H. (2017). Liposomes: Methods and Protocols.

[152] Xiang B., Cao D.-Y., Lu W.-L., Qi X.-R. (2017). Preparation of Drug Liposomes by Thin-Film Hydration and Homogenization. Liposome-Based Drug Delivery Systems.

[153] Ong S.G.M., Chitneni M., Lee K.S., Ming L.C., Yuen K.H. (2016). Evaluation of Extrusion Technique for Nanosizing Liposomes. Pharmaceutics.

[154] Tian T., Li X., Zhang J. (2019). MTOR Signaling in Cancer and MTOR Inhibitors in Solid Tumor Targeting Therapy. Int. J. Mol. Sci..

[155] Marquard F.E., Jücker M. (2020). PI3K/AKT/MTOR Signaling as a Molecular Target in Head and Neck Cancer. Biochem. Pharmacol..

[156] Casadó A., Sagristá M.L., Mora M. (2018). A Novel Microfluidic Liposomal Formulation for the Delivery of the SN-38 Camptothecin: Characterization and in vitro Assessment of Its Cytotoxic Effect on Two Tumor Cell Lines. Int. J. Nanomed..

[157] Zhang M., Wang Y., He Y., Wang H., Chen B., Tu B., Zhu S., Huang Y. (2020). Anti-PD-L1 Mediating Tumor-Targeted Codelivery of Liposomal Irinotecan/JQ1 for Chemo-Immunotherapy. Acta Pharmacol. Sin..

[158] Xing J., Zhang X., Wang Z., Zhang H., Chen P., Zhou G., Sun C., Gu N., Ji M. (2019). Novel Lipophilic SN38 Prodrug Forming Stable Liposomes for Colorectal Carcinoma Therapy. Int. J. Nanomed..

[159] Huang J.-R., Lee M.-H., Li W.-S., Wu H.-C. (2019). Liposomal Irinotecan for Treatment of Colorectal Cancer in a Preclinical Model. Cancers.

[160] Montoto S.S., Muraca G., Ruiz M.E. (2020). Solid Lipid Nanoparticles for Drug Delivery: Pharmacological and Biopharmaceutical Aspects. Front. Mol. Biosci..

[161] Üner M., Yener G. (2007). Importance of Solid Lipid Nanoparticles (SLN) in Various Administration Routes and Future Perspectives. Int. J. Nanomed..

[162] López-García R., Ganem-Rondero A. (2015). Solid Lipid Nanoparticles (SLN) and Nanostructured Lipid Carriers (NLC): Occlusive Effect and Penetration Enhancement Ability. J. Cosmet. Dermatol. Sci. Appl..

[163] Smith T., Affram K., Nottingham E.L., Han B., Amissah F., Krishnan S., Trevino J., Agyare E. (2020). Application of Smart Solid Lipid Nanoparticles to Enhance the Efficacy of 5-Fluorouracil in the Treatment of Colorectal Cancer. Sci. Rep..

[164] Zielińska A., Carreiró F., Oliveira A.M., Neves A., Pires B., Venkatesh D.N., Durazzo A., Lucarini M., Eder P., Silva A.M. (2020). Polymeric Nanoparticles: Production, Characterization, Toxicology and Ecotoxicology. Molecules.

[165] Schaffazick S.R., Pohlmann A.R., Dalla-Costa T., Guterres S.S. (2003). Freeze-Drying Polymeric Colloidal Suspensions: Nanocapsules, Nanospheres and Nanodispersion. A Comparative Study. Eur. J. Pharm. Biopharm..

[166] García M.C. (2020). Nano-and microparticles as drug carriers. Engineering Drug Delivery Systems.

[167] Lu X.-Y., Wu D.-C., Li Z.-J., Chen G.-Q. (2011). Polymer Nanoparticles. Prog. Mol. Biol. Transl. Sci..

[168] Tan C., Arshadi M., Lee M.C., Godec M., Azizi M., Yan B., Eskandarloo H., Deisenroth T.W., Darji R.H., Pho T.V. (2019). A Robust Aqueous Core–Shell–Shell Coconut-like Nanostructure for Stimuli-Responsive Delivery of Hydrophilic Cargo. ACS Nano.

[169] Qian K., Wu J., Zhang E., Zhang Y., Fu A. (2015). Biodegradable Double Nanocapsule as a Novel Multifunctional Carrier for Drug Delivery and Cell Imaging. Int. J. Nanomed..

[170] Johnston A.P., Cortez C., Angelatos A.S., Caruso F. (2006). Layer-by-Layer Engineered Capsules and Their Applications. Curr. Opin. Colloid Interface Sci..

[171] George A., Shah P.A., Shrivastav P.S. (2019). Natural Biodegradable Polymers Based Nano-Formulations for Drug Delivery: A Review. Int. J. Pharm..

[172] Englert C., Brendel J.C., Majdanski T.C., Yildirim T., Schubert S., Gottschaldt M., Windhab N., Schubert U.S. (2018). Pharmapolymers in the 21st Century: Synthetic Polymers in Drug Delivery Applications. Prog. Polym. Sci..

[173] Kapoor D.N., Bhatia A., Kaur R., Sharma R., Kaur G., Dhawan S. (2015). PLGA: A Unique Polymer for Drug Delivery. Ther. Deliv..

[174] Catalin Balaure P., Mihai Grumezescu A. (2015). Smart Synthetic Polymer Nanocarriers for Controlled and Site-Specific Drug Delivery. Curr. Top. Med. Chem..

[175] Osorio M., Martinez E., Naranjo T., Castro C. (2020). Recent Advances in Polymer Nanomaterials for Drug Delivery of Adjuvants in Colorectal Cancer Treatment: A Scientific-Technological Analysis and Review. Molecules.

[176] Luss A.L., Kulikov P.P., Romme S.B., Andersen C.L., Pennisi C.P., Docea A.O., Kuskov A.N., Velonia K., Mezhuev Y.O., Shtilman M.I. (2018). Nanosized Carriers Based on Amphiphilic Poly-N-Vinyl-2-Pyrrolidone for Intranuclear Drug Delivery. Nanomedicine.

[177] Piñón-Castillo H.A., Martínez-Chamarro R., Reyes-Martínez R., Salinas-Vera Y.M., Manjarrez-Nevárez L.A., Muñoz-Castellanos L.N., López-Camarillo C., Orrantia-Borunda E. (2021). Palladium Nanoparticles Functionalized with PVP-Quercetin Inhibits Cell Proliferation and Activates Apoptosis in Colorectal Cancer Cells. Appl. Sci..

[178] Radu I.C., Hudita A., Zaharia C., Galateanu B., Iovu H., Tanasa E., Georgiana Nitu S., Ginghina O., Negrei C., Tsatsakis A. (2019). Poly (3-Hydroxybutyrate-CO-3-Hydroxyvalerate) PHBHV Biocompatible Nanocarriers for 5-FU Delivery Targeting Colorectal Cancer. Drug Deliv..

[179] Handali S., Moghimipour E., Rezaei M., Saremy S., Dorkoosh F.A. (2019). Co-Delivery of 5-Fluorouracil and Oxaliplatin in Novel Poly (3-Hydroxybutyrate-Co-3-Hydroxyvalerate Acid)/Poly (Lactic-Co-Glycolic Acid) Nanoparticles for Colon Cancer Therapy. Int. J. Biol. Macromol..

[180] Wu P., Zhu H., Zhuang Y., Sun X., Gu N. (2020). Combined Therapeutic Effects of 131I-Labeled and 5Fu-Loaded Multifunctional Nanoparticles in Colorectal Cancer. Int. J. Nanomed..

[181] Wu P., Zhou Q., Zhu H., Zhuang Y., Bao J. (2020). Enhanced Antitumor Efficacy in Colon Cancer Using EGF Functionalized PLGA Nanoparticles Loaded with 5-Fluorouracil and Perfluorocarbon. BMC Cancer.

[182] Shad P.M., Karizi S.Z., Javan R.S., Mirzaie A., Noorbazargan H., Akbarzadeh I., Rezaie H. (2020). Folate Conjugated Hyaluronic Acid Coated Alginate Nanogels Encapsulated Oxaliplatin Enhance Antitumor and Apoptosis Efficacy on Colorectal Cancer Cells (HT29 Cell Line). Toxicology.

[183] Yang H., Liu Y., Qiu Y., Ding M., Zhang Y. (2019). MiRNA-204-5p and Oxaliplatin-Loaded Silica Nanoparticles for Enhanced Tumor Suppression Effect in CD44-Overexpressed Colon Adenocarcinoma. Int. J. Pharm..

[184] Cheng G., Zhang X., Chen Y., Lee R.J., Wang J., Yao J., Zhang Y., Zhang C., Wang K., Yu B. (2019). Anticancer Activity of Polymeric Nanoparticles Containing Linoleic Acid-SN38 (LA-SN38) Conjugate in a Murine Model of Colorectal Cancer. Colloids Surf. B Biointerfaces.

[185] Salmanpour M., Yousefi G., Samani S.M., Mohammadi S., Anbardar M.H., Tamaddon A. (2019). Nanoparticulate Delivery of Irinotecan Active Metabolite (SN38) in Murine Colorectal Carcinoma through Conjugation to Poly (2-Ethyl 2-Oxazoline)-b-Poly (L-Glutamic Acid) Double Hydrophilic Copolymer. Eur. J. Pharm. Sci..

[186] Abbasi E., Aval S.F., Akbarzadeh A., Milani M., Nasrabadi H.T., Joo S.W., Hanifehpour Y., Nejati-Koshki K., Pashaei-Asl R. (2014). Dendrimers: Synthesis, Applications, and Properties. Nanoscale Res. Lett..

[187] Alibolandi M., Taghdisi S.M., Ramezani P., Shamili F.H., Farzad S.A., Abnous K., Ramezani M. (2017). Smart AS1411-Aptamer Conjugated Pegylated PAMAM Dendrimer for the Superior Delivery of Camptothecin to Colon Adenocarcinoma in vitro and in Vivo. Int. J. Pharm..

[188] Zhang L., Chan J.M., Gu F.X., Rhee J.-W., Wang A.Z., Radovic-Moreno A.F., Alexis F., Langer R., Farokhzad O.C. (2008). Self-Assembled Lipid− Polymer Hybrid Nanoparticles: A Robust Drug Delivery Platform. ACS Nano.

[189] Zhang L., Zhang L. (2010). Lipid–Polymer Hybrid Nanoparticles: Synthesis, Characterization and Applications. Nano Life.

[190] Dave V., Tak K., Sohgaura A., Gupta A., Sadhu V., Reddy K.R. (2019). Lipid-Polymer Hybrid Nanoparticles: Synthesis Strategies and Biomedical Applications. J. Microbiol. Methods.

[191] Chan J.M., Zhang L., Yuet K.P., Liao G., Rhee J.-W., Langer R., Farokhzad O.C. (2009). PLGA–Lecithin–PEG Core–Shell Nanoparticles for Controlled Drug Delivery. Biomaterials.

[192] Kadletz L., Heiduschka G., Domayer J., Schmid R., Enzenhofer E., Thurnher D. (2015). Evaluation of Spheroid Head and Neck Squamous Cell Carcinoma Cell Models in Comparison to Monolayer Cultures. Oncol. Lett..

[193] Pampaloni F., Reynaud E.G., Stelzer E.H. (2007). The Third Dimension Bridges the Gap between Cell Culture and Live Tissue. Nat. Rev. Mol. Cell Biol..

[194] Kapałczyńska M., Kolenda T., Przybyła W., Zajączkowska M., Teresiak A., Filas V., Ibbs M., Bliźniak R., Łuczewski Ł., Lamperska K. (2018). 2D and 3D Cell Cultures–a Comparison of Different Types of Cancer Cell Cultures. Arch. Med. Sci. AMS.

[195] Kimlin L.C., Casagrande G., Virador V.M. (2013). In vitro Three-dimensional (3D) Models in Cancer Research: An Update. Mol. Carcinog..

[196] Costa E.C., Moreira A.F., de Melo-Diogo D., Gaspar V.M., Carvalho M.P., Correia I.J. (2016). 3D Tumor Spheroids: An Overview on the Tools and Techniques Used for Their Analysis. Biotechnol. Adv..

[197] Liu X., Raju P., Moo-Young M. (2011). In vitro Cancer Model for Drug Testing. Comprehensive Biotechnology. Vol 5: Medical Biotechnology and Healthcare.

[198] Hirschhaeuser F., Menne H., Dittfeld C., West J., Mueller-Klieser W., Kunz-Schughart L.A. (2010). Multicellular Tumor Spheroids: An Underestimated Tool Is Catching up Again. J. Biotechnol..

[199] Goodman T.T., Ng C.P., Pun S.H. (2008). 3-D Tissue Culture Systems for the Evaluation and Optimization of Nanoparticle-Based Drug Carriers. Bioconjug. Chem..

[200] Trédan O., Galmarini C.M., Patel K., Tannock I.F. (2007). Drug Resistance and the Solid Tumor Microenvironment. J. Natl. Cancer Inst..

[201] Smith T., Affram K., Bulumko E., Agyare E. (2018). Evaluation of In-Vitro Cytotoxic Effect of 5-FU Loaded-Chitosan Nanoparticles against Spheroid Models. J. Nat. Sci..

[202] Tchoryk A., Taresco V., Argent R.H., Ashford M., Gellert P.R., Stolnik S., Grabowska A., Garnett M.C. (2019). Penetration and Uptake of Nanoparticles in 3D Tumor Spheroids. Bioconjug. Chem..

[203] Bauleth-Ramos T., Feijão T., Gonçalves A., Shahbazi M.-A., Liu Z., Barrias C., Oliveira M.J., Granja P., Santos H.A., Sarmento B. (2020). Colorectal Cancer Triple Co-Culture Spheroid Model to Assess the Biocompatibility and Anticancer Properties of Polymeric Nanoparticles. J. Control. Release.

[204] Cekanova M., Rathore K. (2014). Animal Models and Therapeutic Molecular Targets of Cancer: Utility and Limitations. Drug Des. Devel. Ther..

[205] Corpet D.E., Pierre F. (2005). How Good Are Rodent Models of Carcinogenesis in Predicting Efficacy in Humans? A Systematic Review and Meta-Analysis of Colon Chemoprevention in Rats, Mice and Men. Eur. J. Cancer.

[206] Cabeza L., Perazzoli G., Mesas C., Jiménez-Luna C., Prados J., Rama A.R., Melguizo C. (2020). Nanoparticles in Colorectal Cancer Therapy: Latest In Vivo Assays, Clinical Trials, and Patents. AAPS PharmSciTech.

[207] Miller C.R., Williams C.R., Buchsbaum D.J., Gillespie G.Y. (2002). Intratumoral 5-Fluorouracil Produced by Cytosine Deaminase/5-Fluorocytosine Gene Therapy Is Effective for Experimental Human Glioblastomas. Cancer Res..

[208] Meng H., Leong W., Leong K.W., Chen C., Zhao Y. (2018). Walking the Line: The Fate of Nanomaterials at Biological Barriers. Biomaterials.

[209] Chenthamara D., Subramaniam S., Ramakrishnan S.G., Krishnaswamy S., Essa M.M., Lin F.-H., Qoronfleh M.W. (2019). Therapeutic Efficacy of Nanoparticles and Routes of Administration. Biomater. Res..

